# Mechanistic insights into solvent-guided growth and structure of MoO_2_ nanoparticles in solvothermal synthesis†

**DOI:** 10.1039/d5sc03247d

**Published:** 2025-06-30

**Authors:** Laura G. Graversen, Mikkel Juelsholt, Olivia Aalling-Frederiksen, Ulrik Friis-Jensen, Rebecca K. Pittkowski, Maria S. Thomsen, Andrea Kirsch, Nicolas P. L. Magnard, Kirsten M. Ø. Jensen

**Affiliations:** a Department of Chemistry and Nano-Science Center, University of Copenhagen 2100 Copenhagen Ø Denmark kirsten@chem.ku.dk; b Research Center Future Energy Materials and Systems of the Research Alliance Ruhr 44801 Bochum Germany; c Faculty of Chemistry and Biochemistry, Ruhr University Bochum 44801 Bochum Germany

## Abstract

Understanding the processes involved in the nucleation and growth of nanoparticles is essential for the development of tailored nanomaterials. Here, we investigate the solvent effects on the atomic structure and size of nanocrystalline MoO_2_ obtained from a solvothermal synthesis and deduce their reaction pathways. Detailed pair distribution function (PDF) analysis reveals the formation of distinct MoO_2_ structures, depending on the alcohol used. We show that the atomic structure and crystallite size of the formed materials are directly related to their formation pathway. *In situ* PDF analysis together with X-ray absorption spectroscopy of the reaction between MoCl_5_ and an alcohol solvent allows us to see that larger nanoparticles (*ca.* 30 nm) with the conventional MoO_2_ distorted rutile structure form when the initial Cl/O-ligand exchange is fast, but the subsequent condensation and crystallization are slowed down in the synthesis process. On the other hand, when the Cl/O exchange is slowed down, a [M^IV^Cl_*x*_O_*y*_]-complex is formed, and we obtain very small nanoparticles (2–3 nm) with the MoO_2_ high-pressure polymorph structure. The study shows how the chemistry of the reaction solvent affects the mechanistic pathways, and consequently the intermediate formed just prior to crystallization, which is directly applicable to the process of obtaining specific nanocrystalline materials.

## Abstract 2

Using *in situ* methods to investigate solvent effects on nanocrystalline MoO_2_ formation, we show that the choice of solvent alters the formation pathway, directly impacting both the atomic structure and crystallite size of the formed materials.

## Graphical abstract

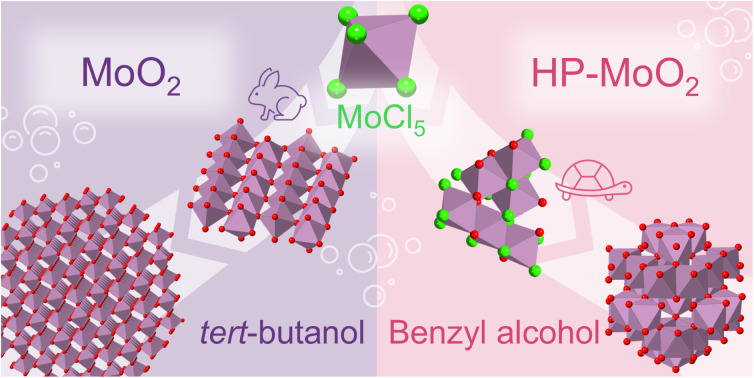

## Introduction

The discovery of new synthetic methods for nanomaterials is largely carried out through time-consuming trial-and-error methods.^1–6^ The reliance on such multiparametric approaches is due to our limited understanding of the underlying chemistry responsible for forming nanomaterials. This knowledge gap slows down material discovery, as the synthetic complexity, along with the infinite combinatory possibilities of elements, makes it impossible to study all the ways nanomaterials with specific properties are produced. To gain insight into the chemistry governing the formation of nanomaterials, it is necessary to understand the material nucleation processes and growth mechanisms. Non-classical nucleation processes that are important in the formation of nanomaterials, include pre-nucleation species that lower the activation energy of material formation.^2,7,8^ It is hypothesized that such intermediates share a local atomic structure with the final product, thus driving the crystallization in one direction or another.^2,9–18^ However, the nucleation processes and the exact role of the intermediate species are not well understood.^2,3,19–22^ Therefore, gaining further understanding of material nucleation and formation is the key to efficiently engineering the synthetic conditions for the desired materials.^1,5,6,23^

**Fig. 1:**
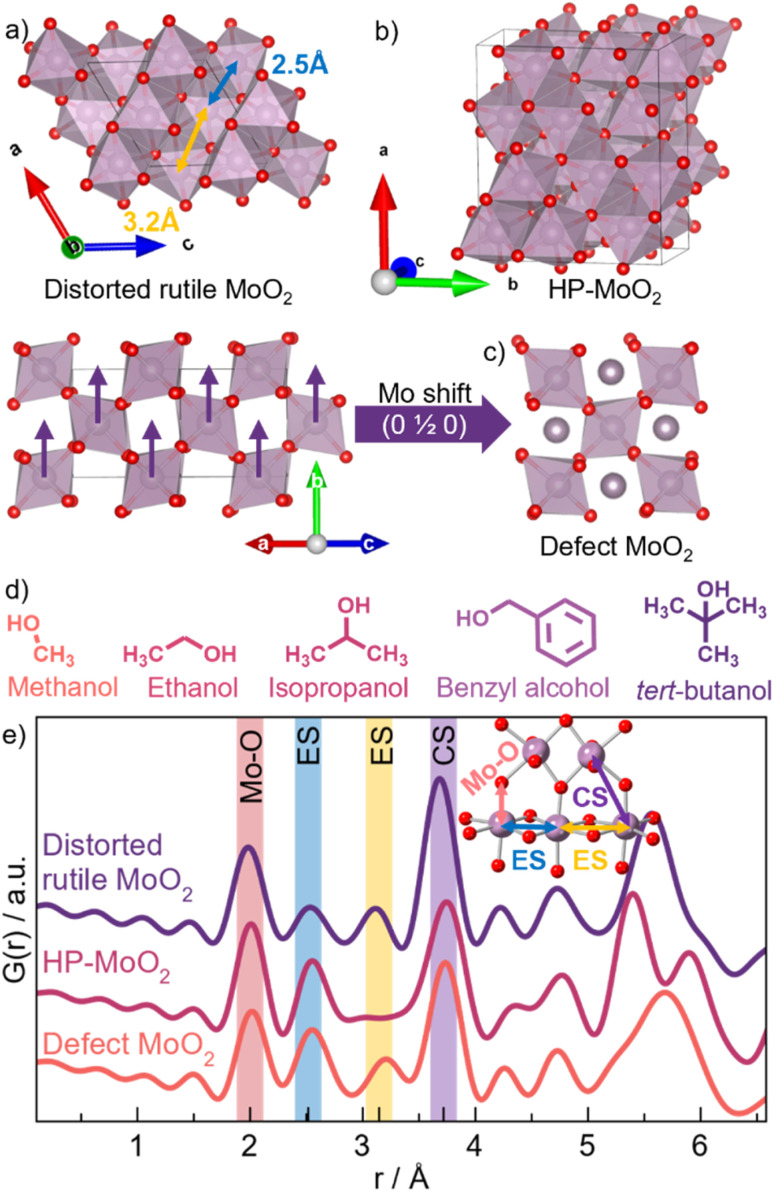
MoO_2_ structures. (a) Distorted rutile MoO_2_. (b) HP-MoO_2_. (c) Defect MoO_2_ structure with additional Mo density in the interstitial sites of the distorted rutile structure. (d) Overview of solvents used. (e) Calculated PDFs from distorted rutile MoO_2_ (purple), HP-MoO_2_ (pink), and defect MoO_2_ (orange) structures.

Here, we investigate the formation of molybdenum oxide nanoparticles, which have gained attention due to their promising applications in catalysis,^24^ energy storage,^25^ and as anode materials in lithium-ion batteries.^25–28^ This wide range of applications stems from the structural diversity and redox chemistry shown by molybdenum-based oxides. Molybdenum(iv) oxide, MoO_2_, crystallizes in a distorted rutile structure (*P*2_1_/*c*) that consists of edge-sharing (ES) chains of [MoO_6_]-octahedra, which are further connected by corner-sharing (CS) oxygen atoms as shown in Fig. 1a.^29^ Spin-pairing of the molybdenum(iv) centers results in an off-centering of the atoms leading to a distortion of the octahedra.^30,31^ Another MoO_2_ polymorph has recently been reported, as Lüdtke *et al.* found that subjecting MoO_2_ to a high pressure of 18 GPa leads to the formation of a high-pressure structure (HP-MoO_2_, *Pnma*) with higher structural complexity than the ambient-pressure counterpart (see Fig. 1b).^32^ The HP-MoO_2_ structure is built from distorted [MoO_6_]-octahedra connected in a corner- and edge-sharing manner, creating alternating zigzagging chains. Furthermore, we have previously observed a significant effect of nanoparticle size on the atomic structure of MoO_2_ nanoparticles.^33^ We discovered that disordered ‘shear planes’ appear in small nanoparticles within the distorted rutile structure, possibly due to surface oxidation. We developed a simple structural model for describing this shear (Fig. 1c), with an interstitial Mo site in the tunnels of the rutile structure (ESI Fig. S1†), as discussed in detail in our previous work. The discovery of new MoO_2_ structures raises key questions about the factors controlling the formation of these metastable phases. In particular, it remains uncertain whether the HP-MoO_2_ phase can be stabilized under conditions far below the high pressures (GPa range) reported to date.

Using molybdenum oxide as a model system, we investigate how synthetic parameters in a straightforward solvothermal synthesis *via* a nonaqueous sol–gel route influence the resulting nanoparticles. By examining the solvent and temperature effects, we aim to understand the correlation between the formation pathway, particle size, and atomic structure. The solvothermal approach is both versatile and environmentally friendly and is widely used for metal oxide nanoparticle production.^34,35^ The nonaqueous sol–gel process involves the reaction between a metal precursor and an organic solvent. In this study, the reaction is between a metal halide and an alcohol that simultaneously acts as the solvent, capping agent, and oxygen supplier.^36,37^ We demonstrate that the choice of alcohol has a significant influence on the material formation pathway, growth, and atomic structure of the resulting particles. We unveil the effects of five different alcohols: methanol, ethanol, isopropanol, benzyl alcohol, and *tert*-butanol, showing that the reactivity of the C–OH bond of the alcohol used is essential in the reaction mechanism.^37–40^ The molecular structures of the alcohols are depicted in Fig. 1d. In addition to parametric studies mapping the size and structure of MoO_2_ nanoparticles, we apply *in situ* X-ray scattering and X-ray absorption spectroscopy (XAS) studies to uncover the reaction pathway and follow the structural changes in the atomic arrangement from the precursors to the final product.^41–47^ From these studies, we show that by controlling the reaction kinetics through temperature and the reactivity of the alcohol, we can select the mechanistic pathway the reaction follows. The pathway directly affects the intermediates produced, thus steering the formation of the desired MoO_2_ material. Employing solvent effects in this manner represents an emerging strategy for synthesizing tailor-made metal oxide nanoparticles through rational synthesis design.

## Experimental methods

### Synthesis of MoO_2_

MoO_2_ samples were synthesized for *ex situ* studies using 0.55 g (0.002 mol) of MoCl_5_ (Sigma-Aldrich, 99.6%) dissolved in 10.0 ml of either methanol, ethanol, isopropanol, benzyl alcohol, or *tert*-butanol in a 23 ml Teflon-lined steel autoclave to obtain a Mo concentration of 0.2 M. The alcohols were used as purchased. The sealed autoclave was placed in a preheated oven at 150 °C or 200 °C for 24 hours. The precipitate was washed with ethanol three times.

### Total scattering and PDF

X-ray total scattering experiments were performed at DESY on beamline P02.1 with an X-ray wavelength of 0.2075 Å^−1^ using a PerkinElmer XRD 1621 flat panel detector.^48^*Ex situ* measurements of MoO_2_ were performed by loading the as-synthesized powders into Kapton tubes with an inner diameter of 1.0 mm. For *in situ* measurements, 0.27 g (0.001 mol) of MoCl_5_ was dissolved in 2.0 ml of the respective alcohol to give a 0.5 M solution. The solution was injected into a custom-made capillary reactor using a setup similar to the one previously described by Becker *et al.* (Fig. S2 in the ESI†).^49^ Fused silica capillaries with 0.7 mm inner diameter and 0.09 mm wall thickness were used. The reactor was pressurized to 100 bar using an HPLC pump. The sample was heated to 150 or 200 °C using a jet of hot air placed below the sample. The X-ray exposure time for each frame was 5 seconds. The obtained data were integrated using PyFAI in Dioptas^50^ and Fourier transformed to PDFs using PDFgetX3 (ref. 51) with *Q*_min_ = 0.5 Å^−1^ and *Q*_max_ = 15 Å^−1^. Subsequent modeling of crystalline structures was performed using PDFgui software.^52^ Modeling of precursor structures and nucleation clusters was performed using Diffpy-CMI software.^53^

### Rietveld refinement of *ex situ* powder X-ray diffraction data

Powder X-ray diffraction (PXRD) data for Rietveld refinement of *ex situ* synthesized MoO_2_ in *tert*-butanol at 200 °C were collected using a Bruker D8 diffractometer in Bragg–Brentano reflection geometry with Cu Kα radiation (*λ* = 1.540 Å). Refinement was performed using FullProf software,^54^ with distorted rutile MoO_2_ as the starting model. A Thompson–Cox–Hastings pseudo-Voigt function was used to model the peak profile, while the background was fitted with a 6th-order Chebyshev polynomial. Lattice parameters, scale factor, thermal vibrations, profile parameters, and zero offset were refined.

### X-ray absorption spectroscopy

XAS was performed in transmission mode at the Mo K-edge (20 000 eV) at the Balder beamline at the MAX IV Laboratory. The measured X-ray absorption near edge structure (XANES) signal ranged from 80 eV below the edge to 200 eV above and was measured using two ion chambers, one placed upstream and one downstream of the sample environment. The extended X-ray absorption fine structure (EXAFS) was measured up to 1000 eV above the absorption edge. The X-ray energy was selected using a double crystal Si(111) monochromator, and prior to each experiment, a piece of molybdenum metal foil was measured for energy calibration. The samples produced *ex situ* were diluted by mixing the powder with polyethylene or boron nitride in a mortar for 20 minutes, pressed into pellets, and enclosed in Kapton films. References were prepared similarly. The optimal dilution ratio to obtain high quality transmission data was estimated using XAFSmass software. *In situ* experiments were performed in our custom-built capillary reactor (Fig. S2 in the ESI†). The reaction mixture was heated by integrated heating filaments to 100, 150, or 200 °C using an external power supply. XAS spectra were collected in continuous scan mode with an acquisition time of 7 seconds in XANES mode and 16 seconds in EXAFS mode. Data treatment was carried out in Athena from the Demeter software package.^55^ The EXAFS Fourier transforms, *χ*(*R*), were obtained from data in the *k*-range of 3–14 Å^−1^, using *k*^2^ weighting, and a Hanning window. Linear combination analysis (LCA) of XANES spectra was carried out over an energy range of 19 985 to 20 050 eV. All phase contents were constrained to values between 0 and 1 and the sum of the phase contents was constrained to 1. The dissolved precursor at room temperature was used as the precursor phase.

## Results and discussion

### Solvent influence on the MoO_2_ structure

**Fig. 2:**
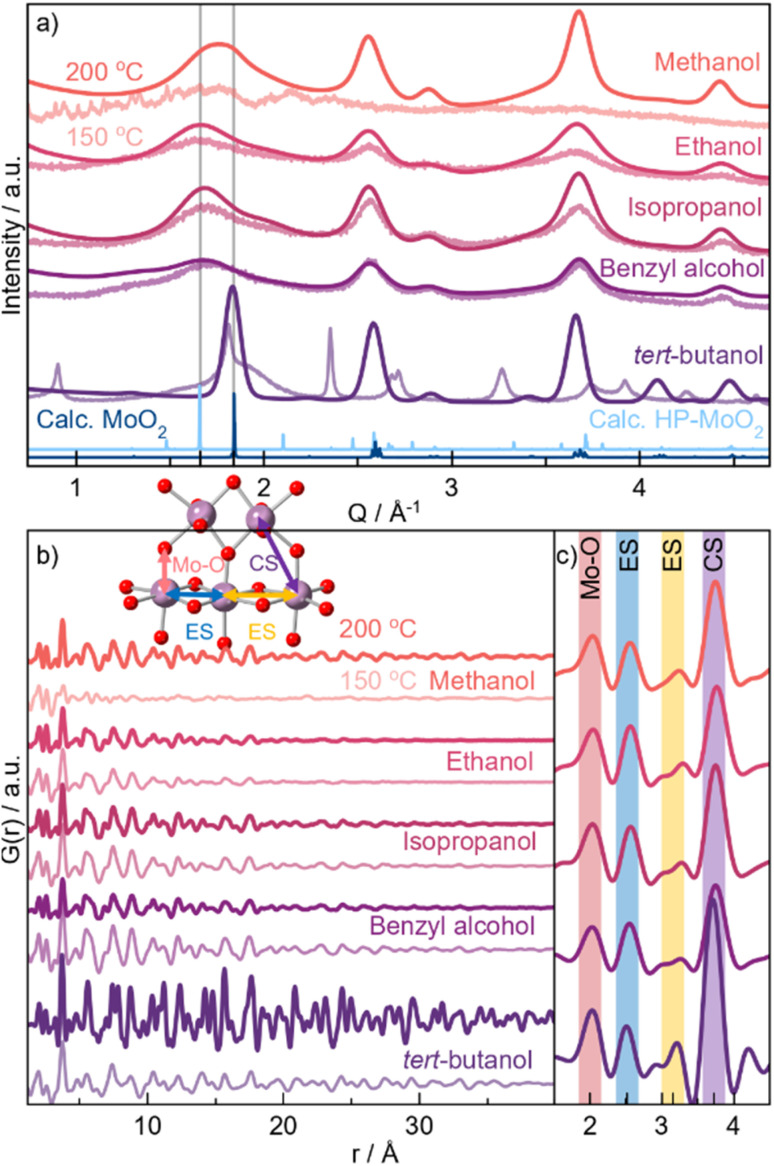
Scattering data for MoO_2_ samples obtained through solvothermal synthesis using five different alcohols and applying temperatures of 150 °C (pale graphs) or 200 °C (dark graphs) for 24 h. (a) Measured PXRD. The two grey lines show the positions of the first observed Bragg peak. PXRD patterns calculated of distorted rutile MoO_2_ (dark blue graph)^29^ and HP-MoO_2_ (light blue graph)^32^ for reference. The data quality for the 200 °C samples is higher due to the formation of larger particles and the greater amount of powder available after synthesis. (b) PDFs obtained for the same samples as in (a). Colored lines represent atomic distances visualized in the inset. (c) Magnification of the local *r*-range in the 200 °C samples.

We first establish the influence of the solvent on the crystal structure of solvothermally synthesized MoO_2_ nanocrystallites using *ex situ* PXRD analysis. The synthesis was carried out using MoCl_5_ with five different alcohols as solvents, with heating at 150 or 200 °C for 24 h. Fig. 2a shows PXRD patterns acquired for the 200 °C samples in dark colors and 150 °C samples in pale. All syntheses performed at 200 °C result in nanoparticles, as observed from the broad Bragg peaks. Comparison with the reference pattern (blue graph) shows that the nanoparticles synthesized in *tert*-butanol at 200 °C adopt the distorted rutile MoO_2_ structure. This is confirmed by Rietveld analysis (Fig. S3†), which shows a crystallite diameter of approx. 30 nm. The Bragg peaks from the other samples are significantly broader, indicating smaller crystallite sizes. Furthermore, the peak positions do not agree with bulk MoO_2_. In particular, the first Bragg peak (1.8 Å^−1^) is shifted to lower *Q*-values compared to the distorted rutile structure, as highlighted by the grey lines in Fig. 2a. This suggests structural deviations from crystalline distorted rutile MoO_2_. The samples treated at 150 °C show similar PXRD patterns for the three syntheses in ethanol, isopropanol, and benzyl alcohol. The reaction in methanol, on the other hand, does not lead to the formation of any crystalline product. An unidentified crystalline phase is formed in *tert*-butanol at 150 °C.

For further structural characterization, we turn to X-ray total scattering and PDF analysis. PDFs from the ten samples are shown in Fig. 2b. We again start by comparing the PDF patterns from the 200 °C syntheses. While the PDF from the *tert*-butanol sample shows clear peaks at *r*-values above 30–40 Å, the correlation length from all other samples is smaller than 30 Å. This corroborates the results from the PXRD analysis. The PDF also shows differences in the atomic structure of the particles. Fig. 2c highlights the local range of the PDFs. Here, we observe a difference in the relative peak intensity between the peak arising from Mo–Mo distances in edge-sharing octahedra (ES, 2.5 Å) and corner-sharing octahedra (CS, 3.6 Å), indicated by the yellow and purple lines in Fig. 2c, respectively. The *tert*-butanol sample has a much more intense CS peak compared to the ES peak. However, the intensity ratio is lowered in the four other samples, *i.e.*, edge-sharing octahedra are more prominent in the other samples with smaller particle sizes. Considering other reported crystal structures for MoO_2_, namely the HP-phase and our previously discussed structural model for small molybdenum oxides, one of the main differences between the structures is exactly the appearance of more [MoO_6_] edge-sharing, as seen from calculated PDFs in Fig. 1e.

**Fig. 3:**
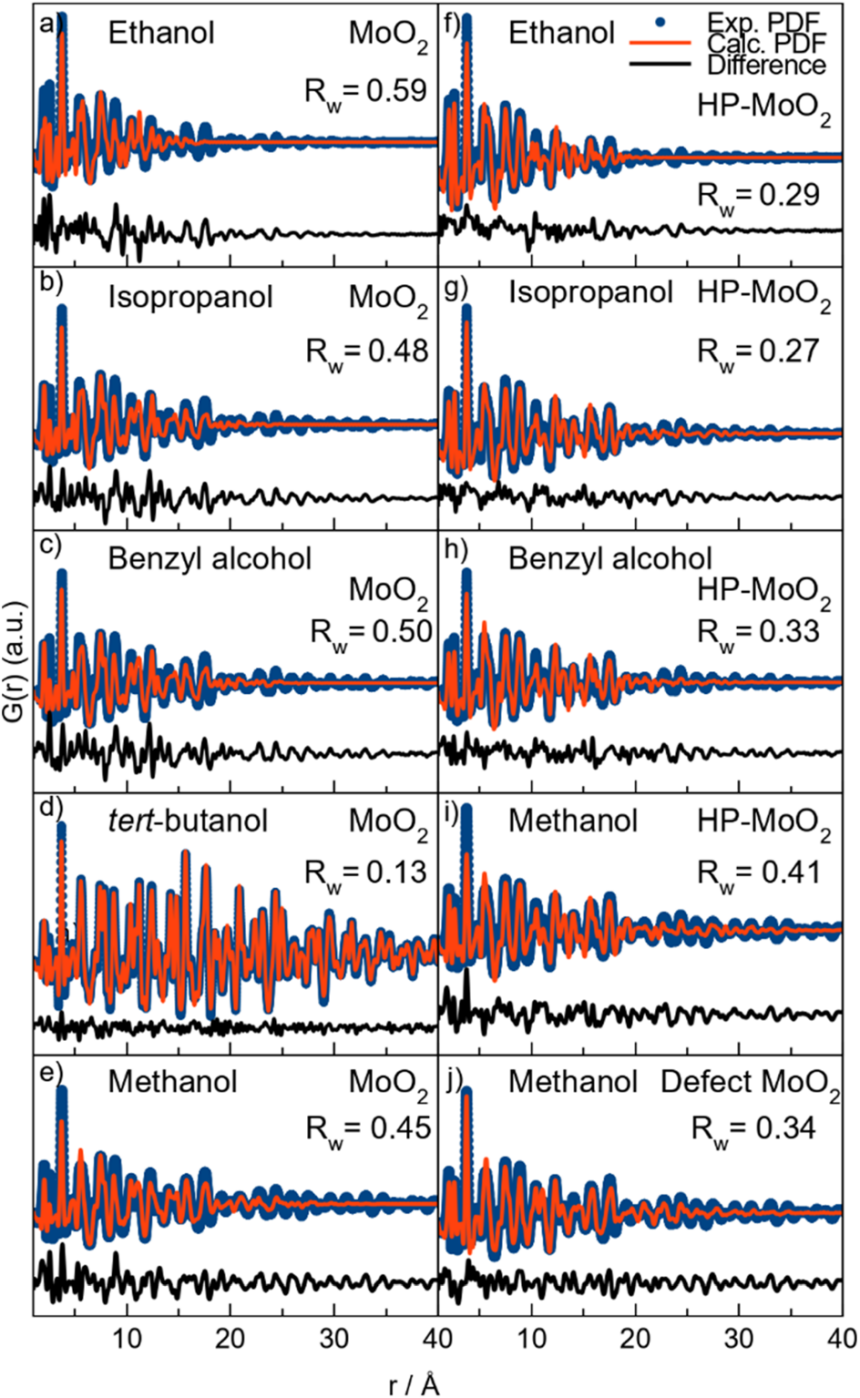
PDFs obtained for MoO_2_ synthesized at 200 °C in (a and f) ethanol, (b and g) isopropanol, (c and h) benzyl alcohol, (d) *tert*-butanol, and (e, i and j) methanol fitted with the distorted rutile MoO_2_ structure and the high-pressure polymorph of MoO_2_ as indicated. (j) Fitted with the distorted rutile MoO_2_ structure with local point defects.

To quantify this, we perform PDF modeling. Fig. 3a–e shows fits with the distorted rutile model to the five samples prepared at 200 °C. As expected, this structure gives a good description of the *tert*-butanol sample. However, the model cannot fully describe the intensity of the PDF peak at 2.5 Å in the other four samples. This highlights that distorted rutile MoO_2_ does not contain enough ES [MoO_6_] to describe the measured data. We therefore test the HP-polymorph, containing zigzagging chains of ES [MoO_6_]-octahedra (Fig. 1b).^32^ Previous studies of nanoparticle structures have shown that very small particles may adopt structures similar to those reported at higher pressure due to the large surface-to-volume ratio of the nanoparticles, which increases the pressure on the particles.^56,57^ Below a certain crystallite size, a transition may occur from energy stabilization primarily dictated by bulk properties to energy destabilization driven by surface effects.^57–59^ Indeed, the HP-model provides an excellent fit to the PDFs of the samples synthesized in ethanol, isopropanol, and benzyl alcohol (see Fig. 3f–h), and the intensity of the ES peak is much better fitted. All refinement results are given in Table S15,† showing particle sizes of 2–3 nm for the three samples. Fits to the samples synthesized at 150 °C are shown in Fig. S8.† These results show that HP-MoO_2_ structured nanoparticles also form at this temperature in ethanol, isopropanol, and benzyl alcohol.

The PDF from the methanol synthesized nanoparticles (Fig. 3i) is not as well described by the HP-phase as the other samples (Fig. 3f–h). Again, relative to the PXRD patterns in Fig. 2a, we also see distinct differences, especially in the first Bragg peak, which is shifted towards lower *Q* values (1.8 Å^−1^). The broad peak is located between that of distorted rutile MoO_2_ in *tert*-butanol, and HP-MoO_2_ in ethanol, isopropanol, and benzyl alcohol. Furthermore, the PDF of the methanol sample shows less ES Mo–Mo peak intensity (blue line in Fig. 2b), compared to that of the ethanol, isopropanol, and benzyl alcohol samples. Neither the distorted rutile MoO_2_ structure nor the HP-MoO_2_ structure gives a satisfactory fit to the data. We therefore tested our previously suggested model for nanostructured MoO_2_ (Fig. 1h), which provides a simplified way of describing disordered shear planes in the rutile structure. Fig. 3j shows how the model improves the fit, compared with the refinements using the distorted rutile (Fig. 3e) or the HP-MoO_2_ (Fig. 3i) structural models. The occupancy of the additional Mo-atoms refines to 30%, showing significant structural changes compared to the distorted rutile structure. The particles synthesized in methanol at 200 °C are larger than those synthesized in ethanol, isopropanol, and benzyl alcohol, with a size of 5 nm. In addition to the different structure, MoO_2_ is not formed in methanol when the temperature is lowered.

Thus, three differently structured MoO_2_ nanoparticles have been successfully synthesized by changing the solvent used in the solvothermal synthesis. The results are summarized in Table 1. In *tert*-butanol, larger particles (*ca.* 30 nm) with the bulk structure, distorted rutile, form at 200 °C, while an unidentified phase appears at lower temperatures. The structure of the 2–4 nm particles formed in ethanol, isopropanol, and benzyl alcohol is best described by the HP-MoO_2_ phase, and their size is dependent on the reaction temperature. In methanol, particles with a defective rutile structure only form at 200 °C. These are slightly larger (*ca.* 5 nm) than those taking the HP-structure. Stabilization of the high-pressure polymorph synthesized in ethanol, isopropanol, and benzyl alcohol may be due to the large surface-to-volume ratio of the 2–4 nm MoO_2_ particles. The 5 nm crystallites formed in methanol appear to be too large to adopt the HP-structure and instead, crystallographic shear planes form within the distorted rutile structure. Given the significance of surface stabilization with a high surface-to-volume ratio, these extended defects could serve as a stabilizing feature to balance the surface energy.

**Table 1:** Overview of crystal structures formed and crystallite sizes obtained from PDF fitting of MoO_2_ formed at 150 or 200 °C using the distorted rutile, HP-MoO_2_, or defect structural model. Full structural details are found in Tables S2–S14 and S16–S18

Solvent	Temperature	Structure	Size (nm)
Methanol	200 °C	Defect	5
	150 °C	—	—
Ethanol	200 °C	HP-MoO_2_	3
	150 °C	HP-MoO_2_	3
Isopropanol	200 °C	HP-MoO_2_	4
	150 °C	HP-MoO_2_	3
Benzyl alcohol	200 °C	HP-MoO_2_	4
	150 °C	HP-MoO_2_	3
*tert*-Butanol	200 °C	Distorted rutile MoO_2_	30
	150 °C	Unidentified	—

Having established that the alcohol used as solvent directly affects the synthesis product, we now consider how the reaction process may take place and how the differences between the reaction products can be explained. Multiple pathways have been suggested for the reaction of a metal halide with an alcohol.^35,37,60^ Generally, the reaction is initiated by the alcohol reacting with the metal precursor, forming either M–OH or M–OR units. These subsequently condense into an extended structure of M–O–M units, which upon further condensation yield the metal oxide structure.^37,60^ At the same time, Mo^5+^ must be reduced to Mo^4+^ to form MoO_2_. The alcohol structure and hence its ability to form a stable carbocation upon substitution, along with its reduction potential, therefore, play an important role in the process. An overview of the physical properties of the alcohols are presented in Table S19 in the ESI.† All alcohols used for synthesis have standard oxidation potentials above *ca.* 2 V, with methanol showing the largest value of 2.73 V_Fc/Fc^+^_, *tert*-butanol 2.60 V_Fc/Fc^+^_, and benzyl alcohol showing the lowest, around 2.0 V_Ag/Ag^+^_. Hence, oxidation potentials do not explain why the different reactions take place.

We therefore consider how the alcohol structure (shown in Fig. 1d) and chemistry might affect the reaction process. *tert*-Butanol is tertiary substituted, isopropanol is secondary substituted, while methanol, ethanol, and benzyl alcohol are primary substituted. This means that the steric hindrance for the reaction to take place differs. The Taft equation (eqn (1)), which is used to estimate reaction rates, can be employed here to quantify the effect of steric hindrance through the Taft steric parameter (*E*_s_).1
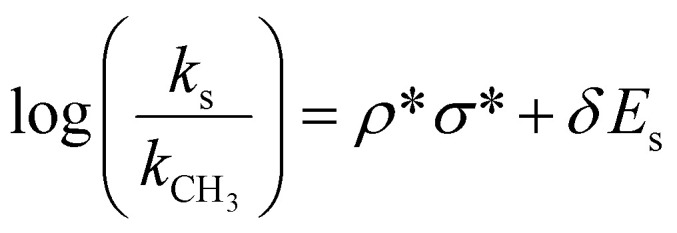
Here, *k*_s_ is the relative difference in the reaction rate of a reaction conducted in a given alcohol compared to a reference reaction in methanol, *k*_CH_3__. ρ* and *δ* are sensitivity factors to polar- and steric effects, respectively, while σ* is the polar substituent constant describing the field and inductive effects. *E*_s_ values for the solvents used are listed in Table 2. A more negative *E*_s_ value leads to a decrease in the reaction rate. The *E*_s_ value for *tert*-butanol is notably more negative compared to the four other alcohols used for synthesis. The bulkiness of the *tert*-butyl group thus results in an elevated energy barrier for particle nucleation. From our *ex situ* results alone, we hypothesize that this is the reason MoO_2_ particles are not formed in *tert*-butanol at 150 °C, while particles form in the less sterically hindered ethanol, isopropanol, and benzyl alcohol. At 200 °C, the slower reaction rate in *tert*-butanol may also facilitate the crystallization of larger nanoparticles adopting the bulk MoO_2_ structure, since the slower reaction prolongs the time before crystallization, meaning prenucleation clusters can form. This leaves more time to build networks of connected [MoO_6_]-octahedra, which eventually link together, resulting in large nanoparticles with the distorted rutile structure. On the other hand, in ethanol, isopropanol, and benzyl alcohol, the solvent chemistry indicates that nucleation is fast, allowing all precursor material to react quickly to form MoO_2_. Therefore, we hypothesize that the fast reaction does not allow enough time for the [MoO_6_]-octahedra to order into the distorted rutile structure.

**Table 2:** Value of the Taft steric parameter *E*_s_ of the substituent attached to –OH^61,62^

R–OH	Methyl	Ethyl	Isopropyl	Benzyl	*tert*-Butyl
*E*_s_	0	−0.07	−0.47	−0.38	−1.54

### *In situ* studies of MoO_2_ formation

To explore the hypotheses regarding reaction rate, crystallization, and solvent chemistry, we now examine the differences in MoO_2_ formation in *tert*-butanol and benzyl alcohol through *in situ* X-ray total scattering. In the subsequent section, we first discuss the experimental observations while relating them to the chemistry of the solvents.

### Formation of distorted rutile MoO_2_ in *tert*-butanol

**Fig. 4:**
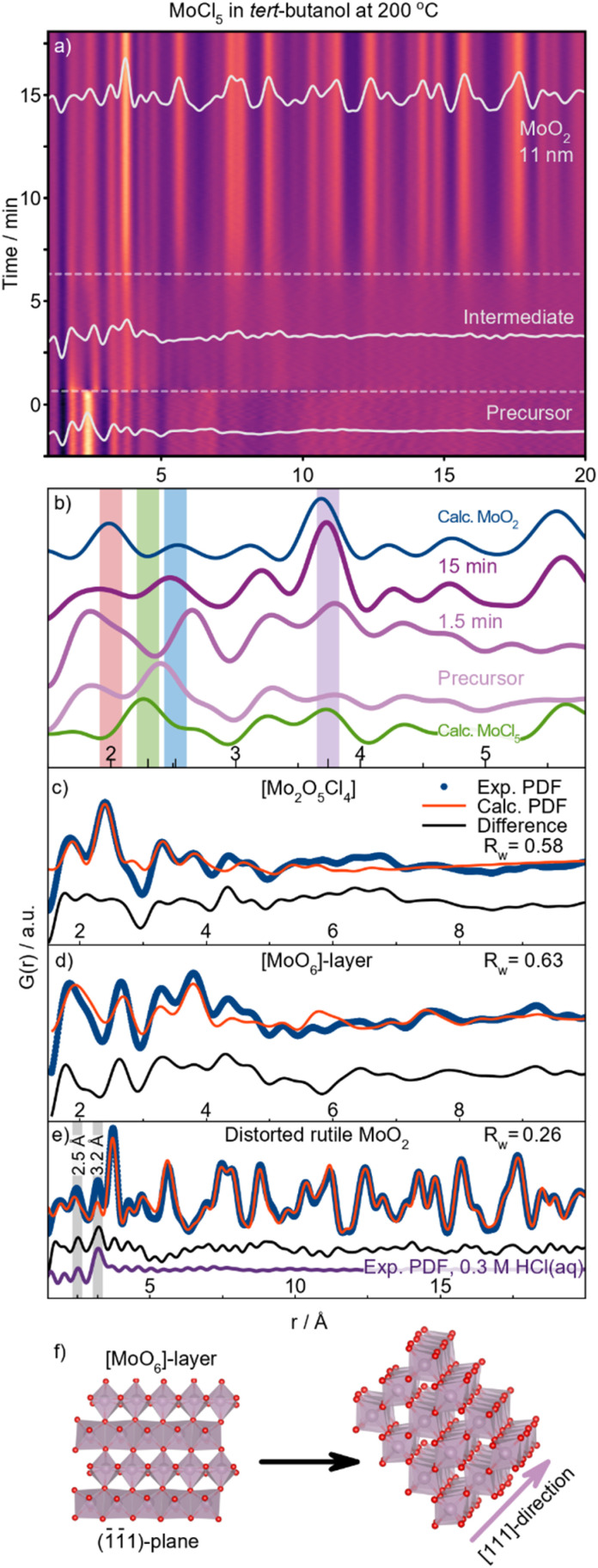
Formation of distorted rutile MoO_2_ in *tert*-butanol at 200 °C. (a) Time-resolved PDFs. 0 minutes designate the time when heating was commenced. (b) Selected PDFs throughout the reaction. (c) Precursor PDF fitted with [Mo_2_O_4_Cl_5_]; (d) intermediate PDF fitted with a layer of [MoO_6_]-octahedra (shown in (f)) cut out from the distorted rutile MoO_2_ structure. (e) PDF fit of the final product using the distorted rutile structure, overlaid with an experimental PDF obtained from 0.3 M HCl_(aq)_. (f) [MoO_6_]-layers assembling along [111].

We start by assessing the reaction pathway for MoO_2_ formation from MoCl_5_ in *tert*-butanol at 200 °C. The time-resolved PDFs are shown in Fig. 4a. The reaction is initiated by heating to 200 °C starting at *t* = 0 min. Inspection of the contour plot indicates a three-phase formation. The PDF from the first stage (marked ‘precursor’ in Fig. 4b) indicates the presence of a chemical species with a short-range ordered structure. We observe clear peaks at *ca. r* = 1.8 Å and 2.4 Å, and smaller peaks extending to 5 Å. While the peak at 1.8 Å corresponds to a Mo–O distance, the peak at 2.4 Å is in agreement with a Mo–Cl distance, as shown in Fig. 4b in comparison with a PDF calculated from the MoCl_5_ crystal structure.^63^ From the PDF, we observe that already at room temperature, some coordinated chloride ligands have been exchanged for oxygen from *tert*-butanol. Similar observations can be drawn from the EXAFS analysis shown in Fig. S10.† The peaks at higher *r*-values (above 3.8 Å) can be assigned to Mo–Mo distances, and their presence shows that the species are not monomeric [MoCl_*x*_O_*y*_]-units, but that a condensation process has taken place. Various [Mo_*z*_O_*x*_Cl_*y*_]-type clusters were used for structural refinement of the precursor PDF (fits in Fig. S11†), where [Mo_2_O_5_Cl_4_] gave the best description of the data (Fig. 4c). We do not assume that this structure uniquely represents the clusters in solution but rather illustrates their main structural motifs. Partial Cl/O-ligand exchange thus occurs at room temperature despite the high steric hindrance of *tert*-butanol, suggesting that this may not be the rate-determining step. We have previously observed similar species as precursors for tungsten oxide and niobium oxides in related syntheses.^64,65^

As soon as heating is initiated, an intermediate species forms (Fig. 4a), exhibiting structural order up to 12 Å. Notably, no Mo–Cl distances are present in the species, as the peak at 2.4 Å disappears immediately upon heating. Comparing the PDFs of the intermediate (1.5 min) and the product (15 min) (Fig. 4b) reveals that peaks from both edge- and corner-sharing [MoO_6_]-octahedra are already present in the intermediate. This shows that similar structural motifs are present in the intermediate species and the final MoO_2_ structure. However, the peak intensity ratios differ from those of the final product. PDF modeling confirms that the PDF is not well described by either of the previously discussed MoO_2_ structures (Fig. S12†). Although the peak positions resemble those in distorted rutile, the slight shifts indicate greater disorder, while the increased intensity of the ES peak at 2.5 Å indicates an ES-rich motif. Therefore, we extracted various structural motifs from the crystalline structure as explained in ESI Fig. S13.† Fitting these different structural motifs, we find that a layer of [MoO_6_]-octahedra cut from the distorted rutile structure along the (1̄1̄1)-plane (Fig. 4f) best describes the intermediate structure (Table S20†). The model gives a reasonable description of the experimental PDF, with all peaks corresponding to the closest atom–atom distance well described (Fig. 4d). We again do not suggest that the model used represents a unique structure solution of the intermediate. Instead, our analysis suggests the structural motif present in the intermediate species.

The intermediate species exists for *ca.* six minutes. As evident from the time-resolved PDFs (Fig. 4a), a sudden formation of a compound with long-range order is then observed. Fig. 4e shows the refinement of the PDF obtained after 15 min using the distorted rutile MoO_2_ structure as the model. As anticipated from our *ex situ* investigations, crystalline particles with the expected bulk structure form. We note that the challenges in describing the 2.5–3.2 Å range likely arise due to the formation of a chlorine hydration shell as further discussed in ESI Fig. S14.† The PDF of aqueous HCl, formed *in situ* according to the proposed reaction mechanisms described below is consistent with the experimental data, showing H–Cl distances at 2.5 and 3.2 Å (grey highlights in Fig. 4e). We hypothesize that the burst formation of MoO_2_ stems from the rapid self-assembly of the intermediate [MoO_6_]-sheets along the [111]-direction, as illustrated in Fig. 4f.

In accordance with the *ex situ* data, no MoO_2_ formation occurs at 150 °C (see ESI Fig. S15†). Nevertheless, shortly after heat is applied, an intermediate species is formed, just as observed in the 200 °C synthesis. The intermediate formed at 150 °C gives rise to PDF peaks at 2.0 Å (Mo–O), 2.5 Å and 3.3 Å (Mo–Mo ES), and 3.6 Å (Mo–Mo CS), resembling the structure of the intermediate formed at 200 °C. However, as evident from Fig. S15c,† difficulties arise in describing the intensity of the Mo–Mo ES peak at 2.5 Å when using the [MoO_6_]-layer model shown in Fig. 4f. This may be due to the trough at 2.9 Å, likely caused by solvation shell scattering. The intermediate is stable at 150 °C as shown by the PDF obtained after 50 min at 150 °C. Hence, the system shows a temperature dependence for overcoming the energy barrier for condensation of the intermediate species, which is insufficient at 150 °C. Our *in situ* studies indicate that the initial Cl/O-ligand exchange is not the rate-determining step in MoO_2_ formation under these conditions, but rather the condensation of the intermediate clusters into crystalline MoO_2_.

### Formation of distorted rutile MoO_2_ in benzyl alcohol

Next, we investigate the formation of MoO_2_ in benzyl alcohol at 200 °C. A contour plot of the PDFs obtained from this reaction is shown in Fig. 7a. Again, we see three stages; a distinct precursor, an intermediate, and a final nanocrystalline material. We start by examining the final structure formed after *t* = 17.5 min. Surprisingly, we observe the formation of the distorted rutile MoO_2_ polymorph (Fig. 7b), *i.e.*, the same product as from *tert*-butanol at the same temperature. As the corresponding autoclave synthesis yielded HP-MoO_2_ (Fig. 1f), this product was not expected. The observed intermediate species appears similar to that seen in the *tert*-butanol synthesis, and can be described using the same [MoO_6_]-sheet (see Fig. 7c). The precursor species, however, differs from the one observed in *tert*-butanol. While the same Mo–Cl and Mo–O distances are observed (see Fig. 7d), the species here appears monomeric, as no peaks corresponding to Mo–Mo distances (3.8 Å) are observed. The structural motif present in the precursor can be represented using the [MoOCl_4_]-unit shown in Fig. 7e. Refinements using other similar structure models are presented in Fig. S16.† Additionally, we performed EXAFS analysis on the precursor solution (Fig. S10a†), which shows the same interatomic distances.

Similar to the synthesis in *tert*-butanol, the Mo–Cl distance at 2.4 Å disappears almost immediately upon heating for 1 min (Fig. 7a). To track and compare the ligand exchange kinetics in benzyl alcohol and *tert*-butanol, a Gaussian function was fitted to the Mo–Cl peak at 2.4 Å. This model-free analysis, presented in Fig. 7a, shows the percentage change in the integrated area of the peak at 2.4 Å. We cannot directly compare the values, but the trend is similar in both solvents, showing a significant decrease in peak intensity after approximately 2 minutes of heating. This again suggests that the activation barrier for Cl/O exchange during the formation of [MoO_*x*_]-sheets is not the rate-determining step at this temperature. However, the intermediate is much shorter-lived in benzyl alcohol, persisting for about 2.5 minutes compared to 6 minutes in *tert*-butanol. As soon as peaks extend beyond 6 Å, condensation of [MoO_*x*_]-clusters begins, which we monitor by fitting a Gaussian function to the CS Mo–Mo peak at 3.6 Å (Fig. 7b). The integrated peak area reflects the number of bonds in the cluster with the respective interatomic distances. In benzyl alcohol (red graph), the CS peak area increases between 2 and 6 minutes, while in *tert*-butanol (purple graph), this increase occurs between 6 and 10 minutes. Although the overall time required for the intermediate clusters to condense into distorted rutile MoO_2_ is similar in both solvents, condensation starts significantly earlier in benzyl alcohol. We relate this difference to a possible stabilizing effect of the [MoO_6_]-sheet from the ligands, as discussed further below.

These *in situ* PDF analyses reveal that the formation of distorted rutile MoO_2_ at 200 °C follows a similar pathway in both benzyl alcohol and *tert*-butanol. Sequential PDF-refinements (shown in ESI Fig. S17†) show that the unit cell contracts along the *a*-direction, while the other unit cell parameters remain relatively stable in both solvents, thus supporting our proposed formation pathway. This behavior indicates that as the disordered sheets assemble along the [111]-direction, structural ordering increases. Additionally, Fig. 7c shows a simultaneous increase in both the crystallite diameter and scale factor in the two experiments, indicating a similar growth mechanism in both solvents through the consumption of precursor in solution. However, crystallites almost twice as large form in *tert*-butanol (11 nm) compared to benzyl alcohol (6 nm), most likely as a result of the delayed intermediate condensation.

### Formation of HP-MoO_2_ in benzyl alcohol

To further investigate the formation process in benzyl alcohol and to search for the previously expected HP-structured material, we performed an experiment at 150 °C. A contour plot of the PDFs is shown in Fig. 7a, where at this lower temperature, HP-MoO_2_ nanoparticles are formed. A fit of the final PDF to the HP-MoO_2_ structure is shown in Fig. 7b, consistent with the results from the autoclave syntheses.

**Fig. 5:**
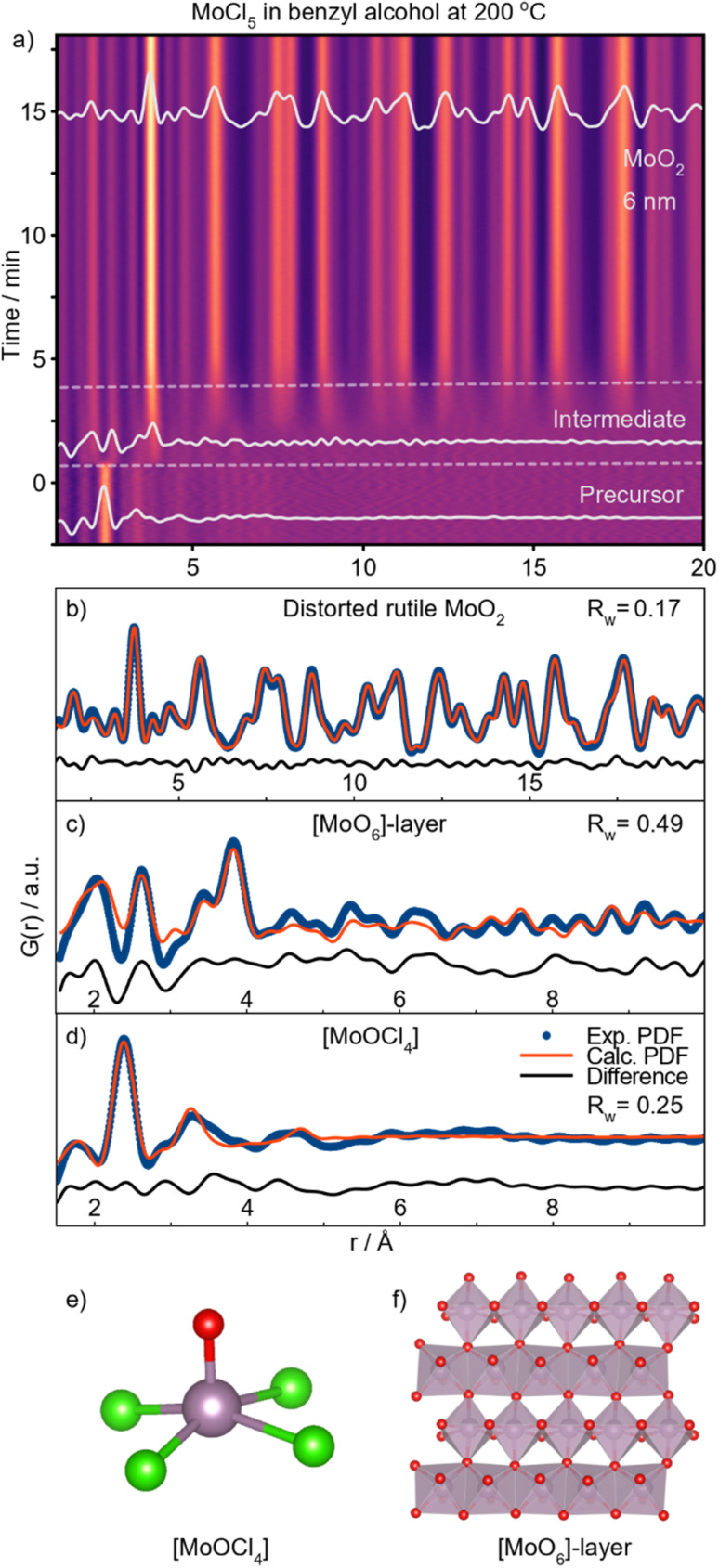
Formation of distorted rutile MoO_2_ using benzyl alcohol as solvent at 200 °C. (a) Time-resolved PDFs. (b) PDF of the final product fitted using distorted rutile MoO_2_. (c) Intermediate fitted with a layer of [MoO_6_]-octahedra (f). (d) Precursor PDF fitted with MoOCl_4_ (e).

The reaction process at 150 °C differs significantly from that at 200 °C (ESI Fig. S18†). At 200 °C, the Mo–Cl peak at 2.4 Å rapidly disappears after heating is started (Fig. 5a), but at 150 °C this peak persists even after 5 minutes (Fig. 7c, green highlight). At this stage, it partly overlaps with the peak originating from ES [MoO_6_] at *r* = 2.5 Å. This suggests that, in contrast to the experiments at 200 °C, the Cl/O-ligand exchange is incomplete at 150 °C, and a [Mo_*z*_O_*x*_Cl_*y*_]-cluster appears to form as an intermediate. We note that we cannot completely rule out a phase mixture of distinct oxy- and chloro-complexes; however, the existence of molybdenum oxochloro compounds supports the formation of a mixed cluster. The integrated area of the Mo–O peak (2.0 Å) gradually increases throughout the reaction at 150 °C (Fig. 7d), reflecting the slow exchange of chloride with oxygen in the intermediate cluster. At the same time, more ES Mo–Mo interactions are incorporated into the cluster, as seen from the increase in peak intensity at 2.5 Å (Fig. 7c, blue highlight). Notably, no slow Cl/O-ligand exchange was observed during the formation of the [MoO_*y*_]_*z*_-intermediate cluster in *tert*-butanol at 150 °C (Fig. 7d), suggesting that two distinct reaction mechanisms occur depending on the alcohol used. The overlap of the Mo–Cl (2.4 Å) and Mo–Mo ES (2.5 Å) peaks in the PDFs prevents tracking of their separate peak intensities. However, the shift in the combined peak position provides insights into the formation kinetics of the intermediate cluster (Fig. 7e, red graph). During the first 15 minutes of the reaction in benzyl alcohol, a distinct shift in PDF peak position from 2.4 Å to 2.5 Å is observed, after which it stabilizes. This shift suggests a reduction in the amount of Mo–Cl pairs (2.4 Å) and an increase in the amount of Mo–Mo ES pairs (2.5 Å) within the [MoO_*x*_Cl_*y*_]_*z*_-intermediate cluster. Simultaneously, a PDF peak representing CS Mo–Mo (at 3.6 Å) is observed at *t* = 12 min (Fig. 7c), which corresponds to formation of corner-sharing [MoO_6_]-octahedra as a result of condensation. At this point, Cl/O-ligand exchange is still taking place, indicating that growth is limited by the condensation of two oxygen-containing nucleation species. Consequently, only small intermediate clusters are formed. Further ordering of this intermediate occurs over time as seen by a sharpening of the peaks, eventually forming HP-MoO_2_ after *t* = 42 min (Fig. 7c). Their small size increases the effective internal pressure on the nanoparticles, possibly contributing to formation of the HP phase, as discussed above.

**Fig. 6:**
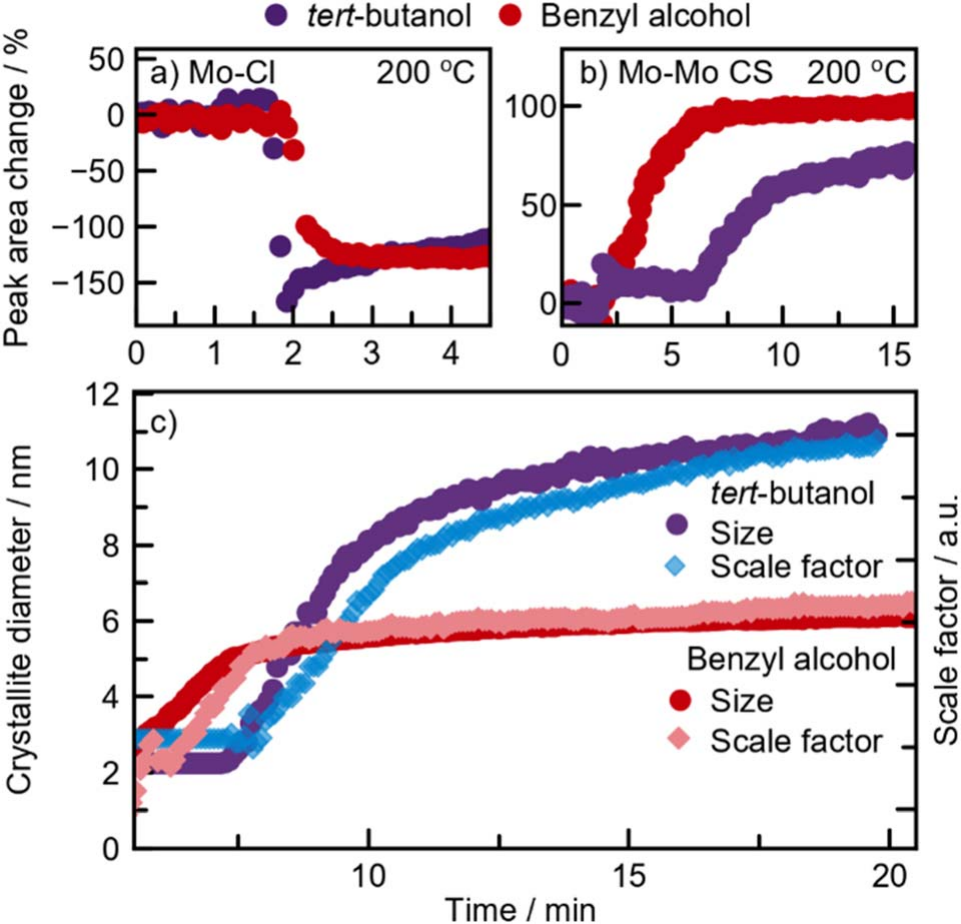
Parameters extracted from *in situ* PDFs of MoO_2_ synthesized at 200 °C in *tert*-butanol and benzyl alcohol. Change of (a) Mo–Cl and (b) Mo–Mo CS peak area obtained from Gaussian fitting. (c) Crystallite sizes and scale factors determined from sequential PDF refinements.

**Fig. 7:**
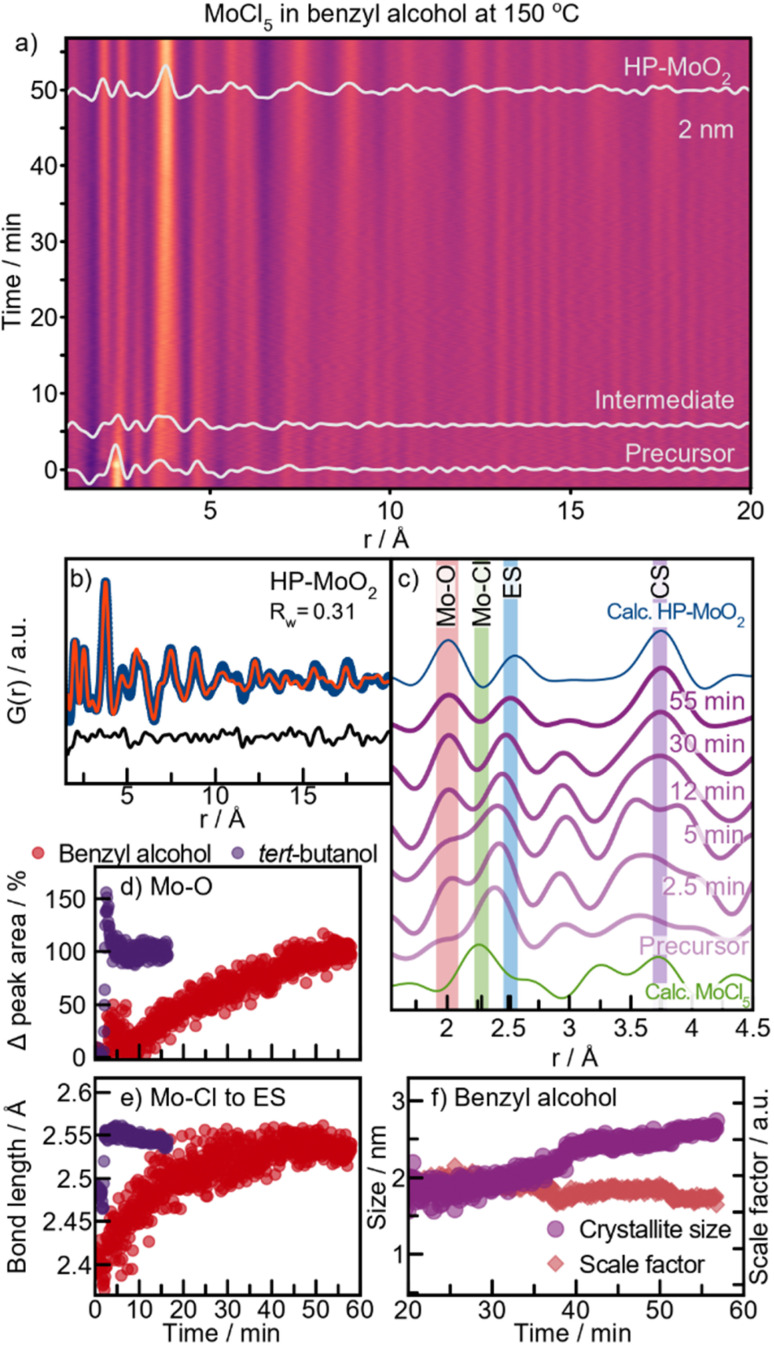
HP-MoO_2_ formation with benzyl alcohol at 150 °C. (a) Time-resolved PDFs. (b) PDF of final product fitted to HP-MoO_2_. (c) Selected PDFs throughout the reaction. (d) Change in the Mo–O peak area and (e) peak position from Gaussian fitting of PDFs during 150 °C syntheses in benzyl alcohol and *tert*-butanol. (f) Crystallite size and scale factor from sequential PDF refinements in benzyl alcohol. Refinement details are provided in Fig. S19.†

The crystallite growth appears to be closely linked to the formation pathway. PDF data show an increase in crystallite size without a corresponding rise in the scale factor, which remains stable from the point where nanocrystalline HP-MoO_2_ is present (Fig. 7f). This result supports our hypothesis that the presence of chloride in the intermediate cluster limits condensation, thus preventing the growth of large particles. Instead, growth likely occurs through mechanisms like Ostwald ripening, where initially formed particles merge into larger crystallites. This growth behavior contrasts with the formation of distorted rutile MoO_2_ at 200 °C, where crystallization is driven by consumption of the precursor in solution (Fig. 6c).

### Investigation of Mo^5+^ reduction with *in situ* X-ray absorption spectroscopy

Our *in situ* PDF studies have elucidated how the nature of the alcohol employed affects the reaction pathway and nucleation time, as well as the atomic structure and size of the product. Before addressing how we may relate the structure of the alcohol to the different reactions, we investigate how the oxidation state of Mo changes during the reactions using *in situ* X-ray absorption spectroscopy (XAS). Through XAS, we can follow the reduction of Mo^5+^ to Mo^4+^ during nucleation in *tert*-butanol and benzyl alcohol, where the alcohol acts as the reducing agent. *In situ* experiments were conducted at 150 °C and 100 °C. This choice is motivated by the observation that the time scales for these experiments are slightly different from those of the *in situ* PDF experiments, as a different setup is used (Fig. S2†). This changes the heating rate of the experiment and the reaction rate. The reactions occur faster in the *in situ* XAS setup when identical temperatures are applied, and we therefore use lower temperatures compared with the *in situ* PDF setup.

**Fig. 8:**
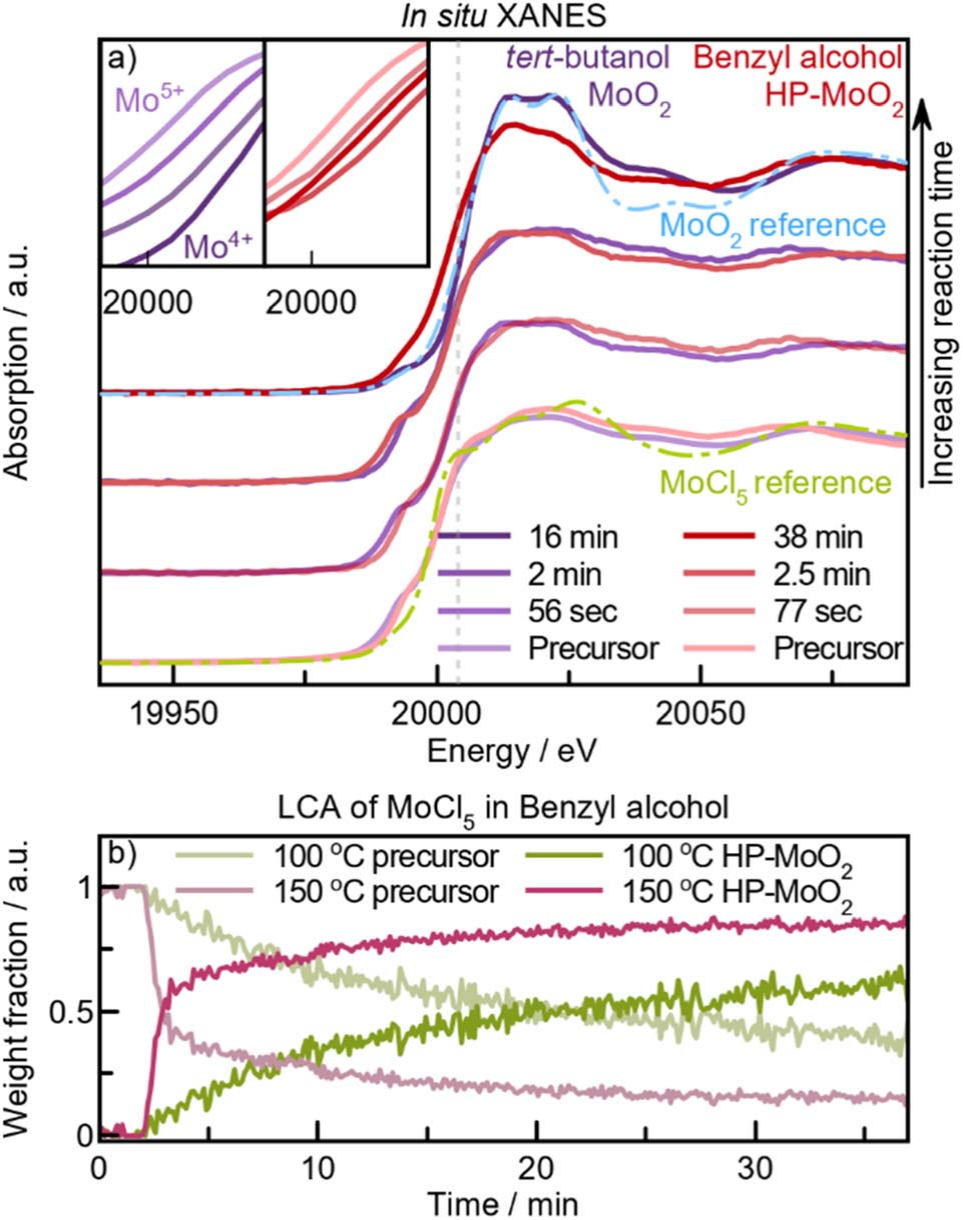
MoO_2_ formation probed by XANES. (a) Solid lines: *in situ* XANES spectra of MoCl_5_ in *tert*-butanol or benzyl alcohol heated to 150 °C at selected time intervals, with the “precursor” at *t* = 0 min. Dashed lines are references: MoCl_5_ (green), MoO_2_ distorted rutile (blue). Vertical grey dashed line is shown as a guide to the eye. Zoomed-in view of the rising edge is shown in the insets. (b) LCA using XANES measured of MoCl_5_ in benzyl alcohol during heating at 100 °C (green) or 150 °C (pink). The measurement at *t* = 0 min is used as precursor weighting, and the XANES spectra measured on *ex situ* produced HP-MoO_2_ as product weighting.

We first consider the product phases observed at the end of the 150 °C experiments, which occur after 16 minutes in *tert*-butanol and 40 minutes in benzyl alcohol (Fig. 8a). From the PDF experiments, we expect these to result in the distorted rutile phase in *tert*-butanol and the HP-MoO_2_ phase in benzyl alcohol. The two absorption spectra do indeed show different shapes of the XANES features, originating from differences in local coordination geometries of the two products, as illustrated in Fig. S20a.† This is further supported by the XANES spectra from the *ex situ* synthesis study (Fig. S20b†), where the *in situ* product from *tert*-butanol shows similar XANES features to the distorted MoO_2_ rutile structure formed *ex situ*, while the *in situ* product formed in benzyl alcohol can be identified as HP-MoO_2_, as expected.

The precursor spectra from the two experiments (at *t* = 0 min) appear similar (Fig. 8a) with white line locations and XANES features resembling those of the MoCl_5_ reference spectrum, indicative of Mo^5+^ in both precursor solutions. As the temperature increases and the intermediates form, the white line moves to higher energies, corresponding to the reduction of Mo^5+^ to Mo^4+^ (Fig. 8a, inset). The white lines of the two product spectra align closely with the MoO_2_ reference, confirming the reduction of Mo^5+^ to Mo^4+^ within 2.5 minutes in both reactions. Full time-resolved spectra are shown in Fig. S21.† However, at this point, pre-edge features at 19 993 eV (*tert*-butanol) and 19 995 eV (benzyl alcohol) are still evident. This suggests that while Mo^5+^ reduction has occurred, the local coordination geometry does not yet resemble the [MoO_6_]-octahedra of MoO_2_. Instead, the local coordination may relate to the precursor species observed in the PDF data for both solvents (Fig. 5d and 7c). The edge location is influenced by both the oxidation state and the ligand environment, as illustrated in Fig. S20a.†

By using Linear Combination Analysis (LCA), we can analyze the reduction kinetics. We here employ a simple approach, using the starting and product XANES spectra as components. This allows determination of the relative proportions of the precursor (Mo^5+^Cl_5−*x*_O_*x*_) and product (Mo^4+^O_2_) present in the reaction mixture. Here, we see that Mo^5+^Cl_5−*x*_O_*x*_ is the dominant species in *tert*-butanol in the first minute of reaction at 150 °C (Fig. S21d†). Subsequently, we see an increase in the Mo^4+^O_2_ component, which becomes dominant after approximately 3 minutes. Between these points, both components are necessary to describe the experimental spectra, again indicating Mo^5+^ to Mo^4+^ reduction occurs within this timeframe. Note that issues with the heater during this experiment cause the oscillations observed in the data. Nevertheless, this observation of a three-phase formation pathway to distorted rutile MoO_2_ aligns with the PDF analysis. Notably, the pre-edge feature of the intermediate XANES spectra (at 19 993 eV, Fig. 8a) cannot be fully described by either the precursor or product components and may be due to the presence of a non-centrosymmetric Mo environment within the intermediate. Combining insights from PDF analysis with changes in the Mo oxidation state, we thus propose that rapid Cl/O-ligand exchange and simultaneous reduction of a Mo^5+^Cl_*x*_O_*y*_ precursor produce a sheet-like Mo^4+^O_*x*_ intermediate, which then condenses into distorted rutile MoO_2_.

Turning to the reduction kinetics governing HP-MoO_2_ formation, the LCA results from the benzyl alcohol experiment (Fig. 8b) reveal a steep decrease in the precursor weight fraction upon heating to 150 °C. Within 3 minutes, the Mo^4+^O_2_ component dominates, which is attributed to the reduction of Mo^5+^ to a Mo^4+^ intermediate, similar to the MoO_2_ formation in *tert*-butanol (Fig. S21d†). Following this rapid rise in the HP-MoO_2_ ratio (at *t* = 3 min), the product fraction continues to increase steadily over time, even as the reaction is terminated. This contrasts with the reaction in *tert*-butanol, indicating a significantly longer transition from the precursor to the product during HP-MoO_2_ formation than during distorted rutile formation.

An additional experiment conducted in benzyl alcohol at 100 °C (green graphs in Fig. 8b), shows a subtle gradual decrease in the precursor component ratio over time. Even after 1 hour of reaction, no MoO_2_ crystallization occurs, as evident from EXAFS (ESI Fig. S22,† “100 °C product” red graph). The presence of oxygen (Mo–O peak at 1.4 Å) and chloride (Mo–Cl peak at 1.9 Å), indicates that a [Mo^5+^Cl_*x*_O_*y*_] species has formed. These gradual changes in the Mo oxidation state evidently prevent crystallite formation, highlighting Mo^4+^ reduction as a key step in the formation pathway of any MoO_2_ polymorph. Combining *in situ* XAS with PDF analysis, we identify two distinct Mo^4+^ intermediates in MoO_2_ formation: [Mo^4+^O_*x*_]_*y*_ leading to distorted rutile, while the proposed [Mo^4+^Cl_*x*_O_*y*_]_*z*_ directs the HP polymorph.

The highly hygroscopic MoCl_5_ precursor is prone to reaction with moisture in the air during sample preparation. Therefore, to determine if this initial oxidation or the possible presence of trace water influences the formation pathway, we conducted *in situ* XANES experiments under various conditions: using non-anhydrous benzyl alcohol and anhydrous benzyl alcohol in atmospheric air as well as in an inert atmosphere. These experiments allowed us to track changes in the oxidation state and composition of the dissolved precursor throughout the solvothermal reaction. Our data (Fig. S23†) showed no significant differences between these experimental conditions, demonstrating that neither trace H_2_O nor aerial oxidation affects the rate of Mo^5+^ reduction. This observation emphasizes that the reduction of Mo^5+^ to Mo^4+^ is a critical step in the formation of MoO_2_, independent of these potential variables.

### Proposed formation processes

Having established the reaction pathway from *in situ* studies, we can now further consider how the reaction may take place. From our *ex situ* studies, as well as previous studies on nonaqueous sol–gel reactions^37,38,40^ as discussed above, steric hindrance was proposed to be the main difference between the reactions in different solvents. However, our *in situ* results show a more complex scenario, as the reaction pathways differ across different solvents.

**Scheme 1:**
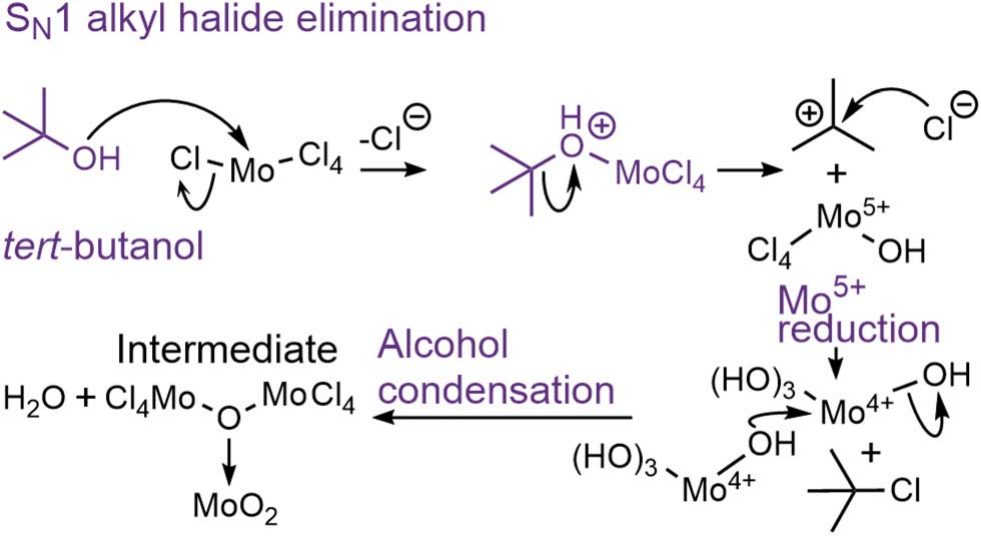
Proposed two-step S_N_1 alkyl halide elimination of *tert*-butyl chloride when MoCl_5_ is solvothermally treated in *tert*-butanol, followed by alcohol condensation. R = H, Mo.

In nonhydrolytic sol–gel synthesis, reaction of metal halides with tertiary species, such as *tert*-butanol, has been described by the groups of Niederberger *et al.*,^37,38^ and Vioux^60,66^ to undergo unimolecular S_N_1 alkyl halide elimination. This two-step reaction is initiated by nucleophilic attack of alcoholic oxygen on the Mo centre upon loss of a Cl^−^ leaving group. The resulting charged species is cleaved, forming Cl_4_MoOH species and strongly inductive-stabilized butyl cations (Scheme 1). This is followed by alcohol condensation of the resulting Mo–OH units into a large MoO_*x*_ network. From our PDF data, regardless of the temperature (Fig. 4 and ESI Fig. S15†), we observe partial burst condensation taking place, showing that the initial Cl/O-ligand exchange is not the limiting factor for reaction. However, the plateauing of the CS Mo–Mo peak intensity (Fig. 6b) between 2.5 and 6 min indicates that the partially condensed intermediate does not continue growing. We hypothesize that the long-lived [Mo^4+^O_*x*_]_*y*_-intermediate (Scheme 1) is surface-stabilized by the *in situ*-generated butyl cations, likely as charged species, preventing full crystallization at 150 °C. At 200 °C, the energy barrier for further condensation is eventually overcome, although the long-lived intermediate appears to direct the formation of large crystallites.

**Scheme 2:**
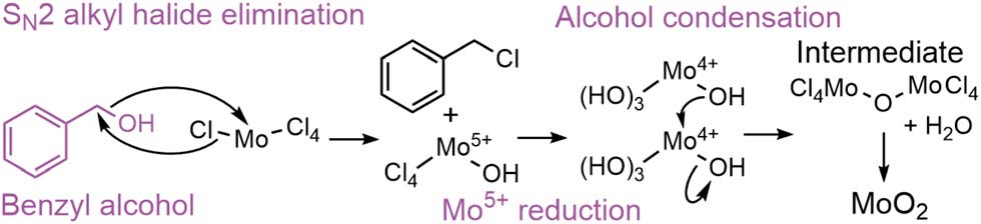
Proposed alcoholysis through S_N_2 alkyl halide formation of MoCl_5_ solvothermally treated in benzyl alcohol followed by alcohol condensation. R = H, Mo.

The nonaqueous sol–gel reaction of benzyl alcohol with metal chloride has been reported to occur through a one-step S_N_2 alkyl halide formation reaction (Scheme 2),^67–72^ producing Cl_4_–Mo–OH units and benzyl chloride.^37,71–73^ In contrast to the reaction in *tert*-butanol, our PDF data (Fig. 7) suggest that in benzyl alcohol, the reaction at low temperature is limited by the initial Cl/O-ligand exchange. This supports a one-step (S_N_2) mechanism in benzyl alcohol, influenced by steric hindrance, compared to the two-step (S_N_1) mechanism in *tert*-butanol, where carbocation stability is essential. At low temperature (150 °C), we see that condensation of the intermediate clusters into CS Mo–Mo octahedra begins before all Cl is exchanged for O, and no ordered intermediate is formed in contrast to the distorted rutile pathway. Instead, a [Mo^4+^Cl_*x*_O_*y*_]_*z*_-intermediate is identified, demonstrating that Cl/O-ligand exchange is the rate-determining step for HP-MoO_2_ formation at low temperatures in benzyl alcohol. At 200 °C, the ligand exchange happens fast, so the ordered rutile-like intermediate forms. It is again the transition from the intermediate to the final MoO_2_ structure that is the rate-limiting step. Here, the PDF data indicate that benzyl chloride stabilizes the intermediate less effectively than butyl cations, resulting in a faster final condensation step in benzyl alcohol than in *tert*-butanol at 200 °C (Fig. 6b). The one-step S_N_2 mechanism in benzyl alcohol enables tuning of the rate-determining step between the Cl/O-ligand exchange (at low temperature) and the intermediate cluster condensation (at high temperature).

**Scheme 3:**
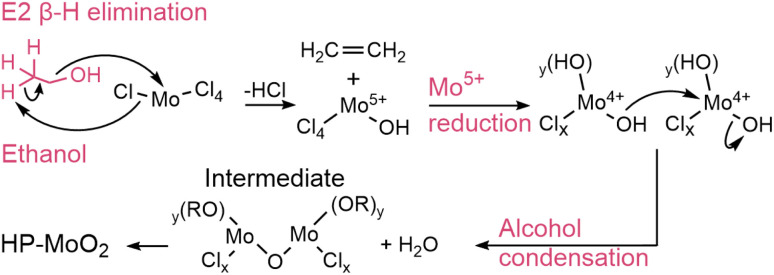
Proposed E2 β-elimination reaction of MoCl_5_ solvothermally treated in ethanol, followed by alcohol condensation. R = H, Mo.

While we do not have *in situ* data for MoO_2_ formation in methanol, ethanol, and isopropanol, we still consider here how their reactions may occur. From our *ex situ* studies, we saw that the reactions in ethanol and isopropanol lead to HP-MoO_2_, *i.e.*, the same product as in benzyl alcohol at low temperature. In methanol, slightly larger particles with a defective rutile-related structure formed. Thus, a distinct reaction pathway may occur with methanol compared to the other solvents. An interesting structural difference between the three primary alcohols is the presence of a β-hydrogen in ethanol and isopropanol, *i.e.*, a hydrogen bound to the second carbon from the nucleophilic OH-group (as illustrated in Scheme 3). This makes an elimination reaction *via* the β-hydrogen possible, which is a common route to metal oxide formation in solvothermal sol–gel syntheses.^37,39,40,74^

**Scheme 4:**
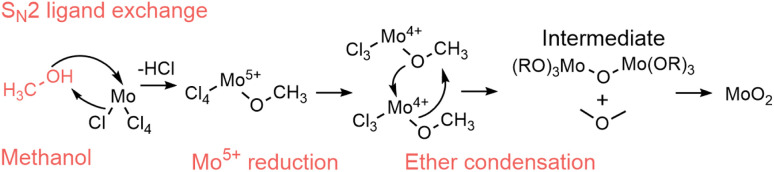
Proposed S_N_2 ligand exchange reaction with HCl formation during the solvothermal treatment of MoCl_5_ in methanol, followed by ether condensation into an extended Mo–O–Mo framework. Dimethyl ether is not experimentally confirmed. R = CH_3_, Mo.

Here, the β-hydrogen facilitates attack by the oxygen lone pair onto Mo supplied by the MoCl_5_ precursor, with elimination of HCl. This alkyl halide elimination may form Cl_4_–Mo–OH units, as verified by cluster fitting of the precursor PDF (Fig. S24†). These units then react *via* alcohol condensation to form a Mo–O–Mo framework that grows until HP-MoO_2_ is finally produced. The lack of a β-hydrogen in methanol favors one-step S_N_2 substitution reactions and is a possible explanation for the different reactions taking place in methanol *versus* ethanol/isopropanol. The ligand exchange in such a reaction progresses through the electrophilic oxygen attacking the nucleophilic Mo-center with Cl^−^ as the leaving group (Scheme 4). The resulting Mo–OR species is believed to undergo ether condensation to form the observed defected MoO_2_. Possibly, this reaction occurs faster than in ethanol and isopropanol, making the particles slightly larger, leading to the defect-rich MoO_2_ rutile structure; however, further *in situ* data would be needed to fully map this process.

**Fig. 9:**
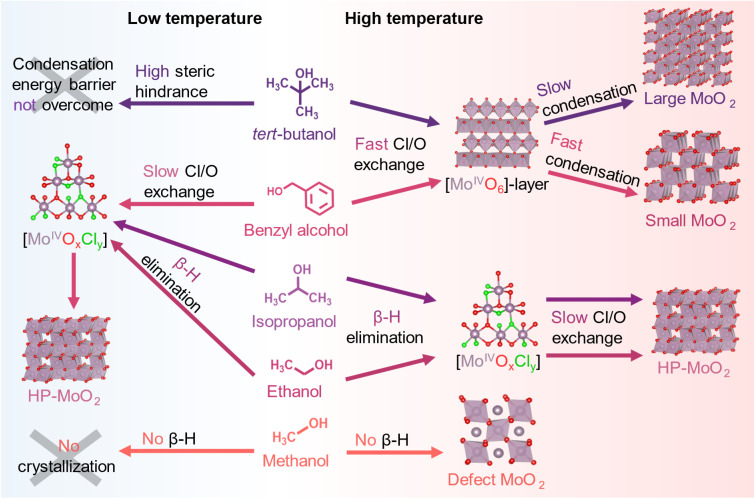
Solvent influence in the solvothermal synthesis of MoO_2_ polymorphs from MoCl_5_ in methanol, ethanol, isopropanol, benzyl alcohol, or *tert*-butanol.

A schematic overview of the solvent influence is shown in Fig. 9.

## Conclusion

The multimodal analysis combining *in situ* PDF and XAS provided valuable insights into the formation mechanism of MoO_2_ nanoparticles. By studying the effects of different solvents in the solvothermal synthesis, three different polymorphs of MoO_2_ were successfully synthesized. The choice of solvent played a crucial role in controlling the size and atomic structure of the nanoparticles, revealing size-structure relationships of the formed nanoparticles.

Specifically, we found that the solvent atomic structure, relating to stability and steric hindrance, affects the reaction pathway and therefore the formed product phase. By carefully controlling the reaction temperature along with the solvent chemistry, the rate-determining step of the formation pathway can be tuned between the ligand exchange (Cl to O) and [MoO_6_] condensation. Generally, we observe that distorted rutile structured MoO_2_ forms if an instantaneous Cl/O-ligand exchange and Mo^5+^ reduction are followed by the formation of [Mo^4+^O_6_]-sheets. These stable intermediate clusters have a structural motif very similar to the atomic structure of the formed distorted rutile MoO_2_ structure. This reaction takes place in both *tert*-butanol and benzyl alcohol at 200 °C, where the initial Cl/O-ligand exchange is fast, but the following condensation of the intermediates is relatively slow. The last step of the reaction appears to be influenced by the nature of the solvent. In *tert*-butanol, crystallization occurs much later than in benzyl alcohol, which we relate to the increased steric hindrance in *tert*-butanol as this raises the energy barrier for condensation and delays crystallization. In *tert*-butanol, protonated *tert*-butyl O–Mo complexes may also stabilize the intermediate sheets. These effects can impede the diffusion-driven reaction between the intermediate clusters, ultimately resulting in larger crystallites in *tert*-butanol (11 nm) compared to those in benzyl alcohol (6 nm) under the same conditions. At a lower reaction temperature of 150 °C, crystallization does not occur in *tert*-butanol. Although Cl/O exchange and the formation of an intermediate molybdenum oxo-species takes place, the temperature is insufficient to overcome the energy barrier required for condensation into crystalline particles.

In benzyl alcohol at 150 °C, complete Cl/O exchange does not occur instantaneously after heating, and ligand exchange appears to be the limiting step. This behavior contrasts with reactions carried out in *tert*-butanol and possibly relates to the different reaction mechanisms expected in the two solvents (S_N_1 *vs.* S_N_2). Condensation at low temperatures in *tert*-butanol is likely hindered by the stabilization of intermediate carbocations, a step absent in the S_N_2 Cl/O-ligand exchange mechanism proposed for benzyl alcohol. During the ligand exchange, a rapid reduction of Mo^5+^ to Mo^4+^ takes place, as observed through *in situ* XANES, forming [Mo^4+^Cl_*x*_O_*y*_] clusters. Cluster condensation into an extended Mo–O–Mo network begins even before the chloride exchange is fully complete, ultimately leading to the formation of HP-MoO_2_. This indicates that the initial ligand exchange step kinetically limits nucleation, preventing the burst nucleation observed at higher temperatures. From *ex situ* studies, we saw that similar products were formed in methanol and ethanol, and we speculate that a similar reaction may occur here; however, further *in situ* studies would be needed to confirm this. Interestingly, the use of methanol as a solvent led to the introduction of point defects embedded in the MoO_2_ structure. This may be related to the absence of a β-hydrogen in methanol, which prohibits O-donation through a β-hydrogen elimination reaction.

PDF analysis allowed us to follow the formation of nanocrystalline molybdenum oxide through two different formation pathways, both starting from MoCl_*x*_O_*y*_ precursor complexes. Here, it is not the structure of the precursor species that is important, but rather the composition of the intermediate species that dictates the atomic structure present in the formed nanocrystallites. Our findings show that mechanistic insights help rationalize which product-directing intermediates might form based on the steric bulkiness of the O-donating solvent. Leveraging solvent effects in this manner is an emerging strategy for synthesizing tailored ultra-small transition metal oxide nanoparticles through rational synthesis design.

## Author contributions

L. G. G., M. J., O. A., and K. M. Ø. J. conceptualized the project. L. G. G. prepared the samples and performed *ex situ* experiments. L. G. G., M. J. and K. M. Ø. J. designed and planned the *in situ* experiments. The *in situ* experiments were performed by L. G. G., U. F., M. S. T., A. K., and N. P. L. M. XAS analysis was performed by L. G. G., U. F., and R. K. P. The original draft was written by L. G. G., M. J., and K. M. Ø. J., with all authors contributing to the review and editing of the paper.

## Conflicts of interest

The authors declare no competing financial interest.

## Supplementary Material

SC-OLF-D5SC03247D-s001

## Data Availability

Data are available from the authors and from https://sid.erda.dk/sharelink/gI71HeJ9u5.
