# Supplementary material for: Mechanistic insights into solvent-guided growth and structure of MoO2 nanoparticles in solvothermal synthesis
Source: Chem Sci. 2025 Jun 30;16(31):14350–65. doi: 10.1039/d5sc03247d (PMC12243927; doi:10.1039/d5sc03247d)
Supplement: SC-016-D5SC03247D-s001 [file SC-016-D5SC03247D-s001.pdf]

Supplementary Information (SI) for Chemical Science.
This journal is © The Royal Society of Chemistry 2025

Supporting information for:

Mechanistic Insights into Solvent-Guided Growth and Structure of MoO_2 Nanoparticles in Solvothermal Synthesis

Laura G. Graversen,[1] Mikkel Juelsholt,[1] Olivia Aalling-Frederiksen,[1] Ulrik Friis-Jensen,[1] Rebecca K. Pittkowski,[1] Maria S. Thomsen,[1] Andrea Kirsch,[1,2,3] Nicolas P. L. Magnard,[1] and Kirsten M. Ø. Jensen*[,1]

[1]Department of Chemistry and Nanoscience Center, University of Copenhagen, 2100 Copenhagen Ø, Denmark

[2]Research Center Future Energy Materials and Systems of the Research Alliance Ruhr, 44801 Bochum, Germany

[3]Faculty of Chemistry and Biochemistry, Ruhr University Bochum, 44801 Bochum, Germany

Contents

Structural model of defect MoO_2 2
In situ setup 2
Refinements of *ex situ* syntheses at 200 °C 3
tert-butanol 3
Methanol 4
Ethanol 5
Isopropanol 6
Refinements of *ex situ* syntheses at 150 °C 9
Examination of physical properties of the alcohols 11
Calculated PDF 12
Investigation of the precursor solutions 12
Formation of distorted rutile MoO_2 in *tert*-butanol 13
Formation of distorted rutile MoO_2 in benzyl alcohol 16
Formation of HP-MoO_2 in benzyl alcohol 17
X-ray absorption spectroscopy 18
XAS study of the possible influence of trace water on Mo^V reduction 20
PDF cluster fits of $MoCl_5$ in ethanol or methanol 21
Crystallographic Information Files reference codes 22
References 22

Structural model of defect MoO_2

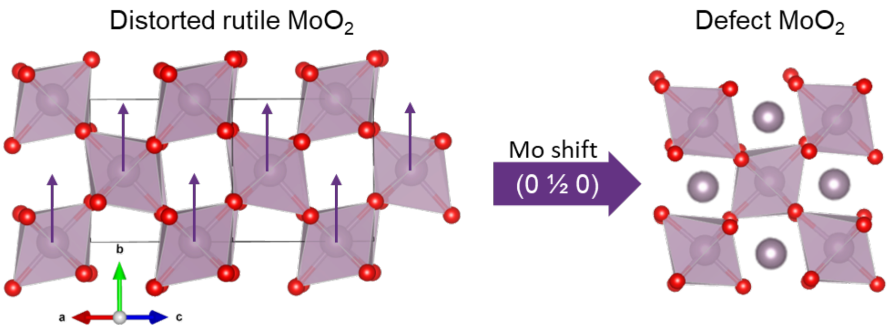

Figure S1. The defect MoO_2 model as created by Lindahl Christiansen et al.[1]

The defect MoO_2 structure is used as a simple model with few extra parameters to describe local point defects created by additional Mo density within the interstitial sites of the distorted rutile MoO_2 structure. The model is created by shifting Mo-atoms by (0, ½, 0) in the distorted rutile structure. Thus creating an interwoven structure of two superimposed distorted rutile structures in the same fcc oxygen lattice. The occupancy of the interstitial Mo atoms is refined to be around 30%. This leads to an increase in edge-sharing Mo-Mo distances observed in the PDF as an increase of the 2.5 Å peak intensity.

In situ setup

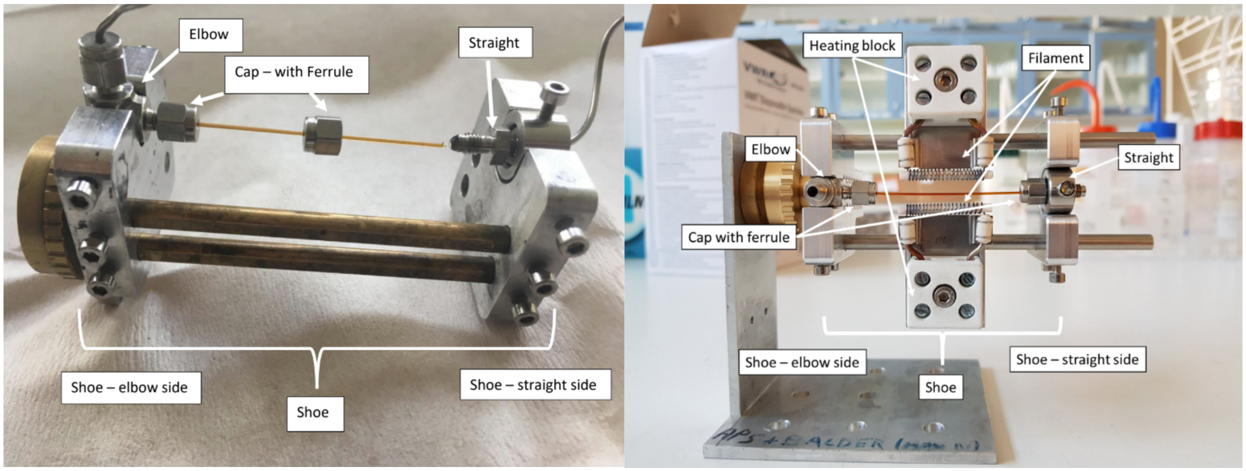

Figure S2. Left) Custom-built capillary reactor used for *in situ* PDF experiments, with heating from below supplied by the "Mini Hot-Air Blower" supplied by beamline P02.1 at DESY. Right) Custom-built capillary reactor with integrated heating filaments used for *in situ* XAS experiments.

Refinements of *ex situ* syntheses at 200 °C

tert-butanol

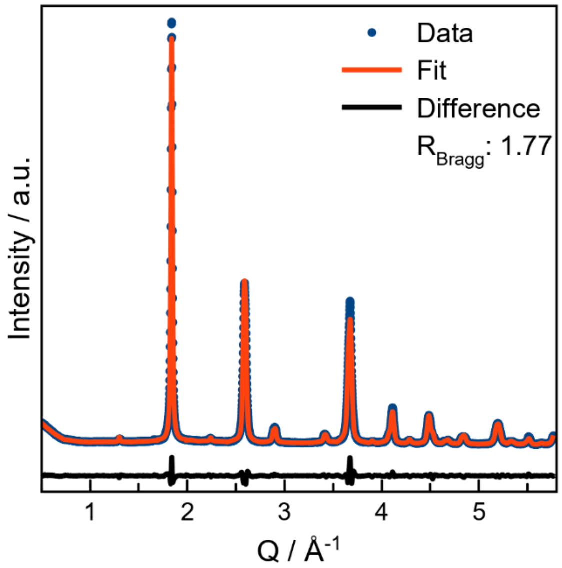

Figure S3. Rietveld refinement of distorted rutile MoO_2 synthesized in *tert*-butanol at 200 °C.

Table S1. Refined parameters from Rietveld refinement of data collected on $MoCl_5$ in *tert*-butanol at 200 °C shown in Figure S3. Distorted rutile MoO_2 was used as the starting model.

**Scale factor**	0.034445122		
**Cell parameters [Å]**	a=5.622328	b=4.845070	c=5.612942
**Cell parameters [º]**		β=120.686661	
**FWHM parameters**	Y= 0.164439	U=0.284495	
**R-values**	R_{wp}= 0.791	R_f=0.138	
**Isotropic crystal size [nm]**	34.2		

Table S2. Refinement parameters from real-space Rietveld refinement shown in Figure 3d. Distorted rutile MoO_2 was used as the structural model, and the data was fitted in a range of 1 Å – 50 Å.

**Scale factor**	**0.5599**		
**Cell parameters [Å]**	a=5.6367	b=4.8617	c=5.5662
**Cell parameters [º]**		β=119.647	
**U_{iso} [$Å^2$]**	Mo=0.00285	O=0.00702	
**δ_2 [$Å^2$]**	1.8		
**sp-diameter [Å]**	$7.481E^{+64}$		
**R_w**	0.13		

Methanol

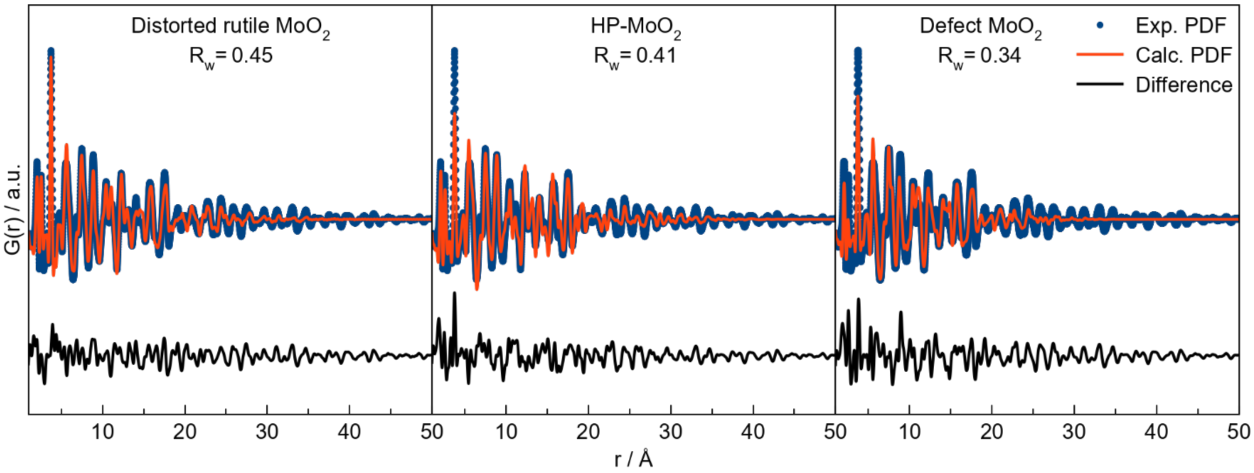

Figure S4. Real-space Rietveld refinement of molybdenum oxide formed *ex situ* in methanol at 200 °C.

Table S3. Refinement parameters from real-space Rietveld refinement shown in Figure S4 left. Distorted rutile MoO_2 was used as the structural model, and the data was fitted in a range of 1 Å – 50 Å.

**Scale factor**	0.4145		
**Cell parameters [Å]**	a=9.8686	b=8.4819	c=4.7719
**U_{iso} [Å^2]**	Mo=0.0104	O=0.0361	
**δ_2 [Å^2]**	2.733		
**sp-diameter [Å]**	50.2191		
**R_w**	0.45		

Table S4. Refinement parameters from real-space Rietveld refinement shown in Figure S4 middle. HP-MoO_2 was used as the structural model, and the data was fitted in a range of 1 Å – 50 Å.

**Scale factor**	0.4929		
**Cell parameters [Å]**	a=9.8737	b=8.4781	c=4.7696
**U_{iso} [Å^2]**	Mo=0.0108	O=0.00938	
**δ_2 [Å^2]**	3.6183		
**sp-diameter [Å]**	48.4494		
**R_w**	0.41		

Table S5. Refinement parameters from real-space Rietveld refinement shown in Figure S4 right. Defect MoO_2 was used as the structural model, and the data was fitted in a range of 1 Å – 50 Å.

**Scale factor**	0.4473		
**Cell parameters [Å]**	a=5.6449	b=4.7634	c=5.7520
**Cell parameters [º]**		β=120.002	
**Defect Mo occupancy**	Mo=0.27		
**U_{iso} [Å^2]**	Mo=0.0104	O=0.0138	
**sratio [Å^2]**	0.05864	rcut=4.0	
**sp-diameter [Å]**	46.7954		
**R_w**	0.34		

Ethanol

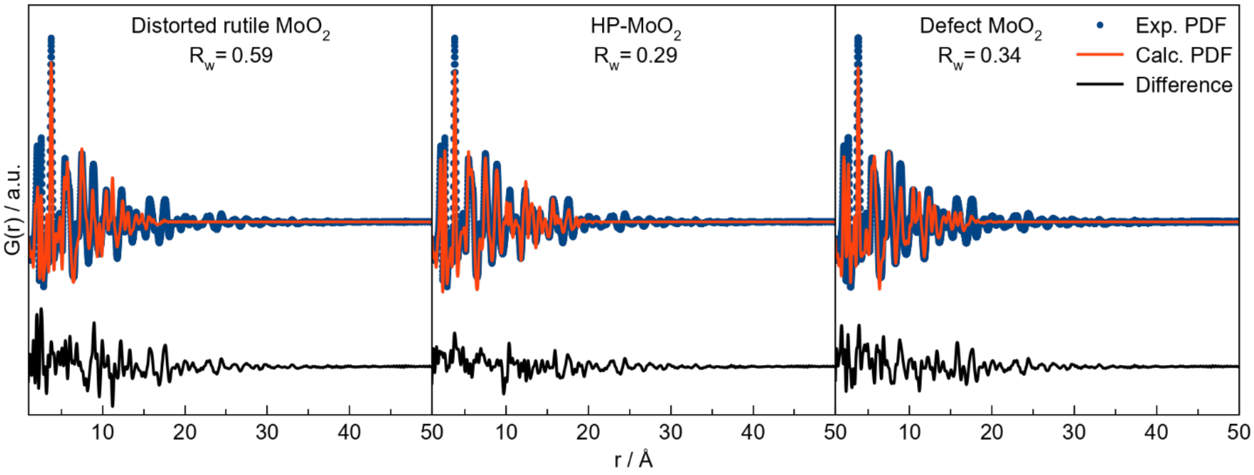

Figure S5. Real-space Rietveld refinement of molybdenum oxide formed *ex situ* in ethanol at 200 °C.

Table S6. Refinement parameters from real-space Rietveld refinement shown in Figure S5 left. Distorted rutile MoO_2 was used as the structural model, and the data was fitted in a range of 1 Å – 50 Å.

**Scale factor**	0.5231		
**Cell parameters [Å]**	a=5.8673	b=4.8253	c=5.6144
**Cell parameters [º]**		β=120.054	
**U_{iso} [Å^2]**	Mo=0.00336	O=0.00161	
**δ_2 [Å^2]**	2.5		
**sp-diameter [Å]**	19.0623		
**R_w**	0.59		

Table S7. Refinement parameters from real-space Rietveld refinement shown in Figure S5 middle. HP-MoO_2 was used as the structural model, and the data was fitted in a range of 1 Å – 50 Å.

**Scale factor**	0.4887		
**Cell parameters [Å]**	a=9.8299	b=8.4944	c=4.814
**U_{iso} [Å^2]**	Mo= 0.0122	O=0.0218	
**sratio [Å^2]**	0.3877	rcut=4.1	
**sp-diameter [Å]**	27.7144		
**R_w**	0.29		

Table S8. Refinement parameters from real-space Rietveld refinement shown in Figure S5 right. Defect MoO_2 was used as the structural model, and the data was fitted in a range of 1 Å – 50 Å.

**Scale factor**	0.5384		
**Cell parameters [Å]**	a=5.6126	b=4.7904	c=5.8857
**Cell parameters [°]**		β=120.093	
**Defect Mo occupancy**	Mo=0.30		
**U_{iso} [Å^2]**	Mo=0.00572	O=0.0473	
**δ_2 [Å^2]**	3.5187		
**sp-diameter [Å]**	21.9722		
**R_w**	0.40		

Isopropanol

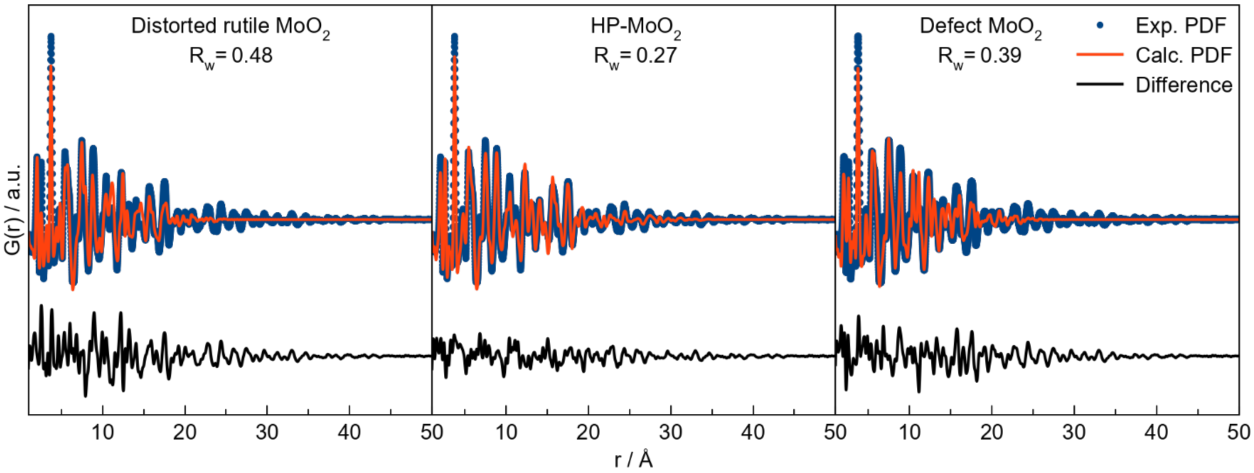

Figure S6. Real-space Rietveld refinement of molybdenum oxide formed *ex situ* in isopropanol at 200 °C.

Table S9. Refinement parameters from real-space Rietveld refinement shown in Figure S6 left. Distorted rutile MoO_2 was used as the structural model, and the data was fitted in a range of 1 Å – 50 Å.

**Scale factor**	0.5123		
**Cell parameters [Å]**	a=5.7292	b=4.8757	c=5.5189
**Cell parameters [°]**		β=119.003	
**U_{iso} [$Å^2$]**	Mo=0.00601	O=0.00586	
**δ_2 [$Å^2$]**	3.6180		
**sp-diameter [Å]**	27.7328		
**R_w**	0.48		

Table S10. Refinement parameters from real-space Rietveld refinement shown in Figure S6 middle. HP-MoO_2 was used as the structural model, and the data was fitted in a range of 1 Å – 50 Å.

**Scale factor**	0.5731		
**Cell parameters [Å]**	a=9.8449	b=8.4872	c=4.7736
**U_{iso} [$Å^2$]**	Mo= 0.00701	O=0.00765	
**sratio [$Å^2$]**	0.4377	rcut=4.0	
**sp-diameter [Å]**	38.8458		
**R_w**	0.27		

Table S11. Refinement parameters from real-space Rietveld refinement shown in Figure S6 right. Defect MoO_2 was used as the structural model, and the data was fitted in a range of 1 Å – 50 Å.

**Scale factor**	0.5714		
**Cell parameters [Å]**	a=5.6365	b=4.7636	c=5.8415
**Cell parameters [°]**		β=120.307	
**Defect Mo occupancy**	Mo=0.30		
**U_{iso} [$Å^2$]**	Mo=0.00522	O=0.0281	
**δ_2 [$Å^2$]**	3.1040		
**sp-diameter [Å]**	29.4077		
**R_w**	0.39		

Benzyl alcohol

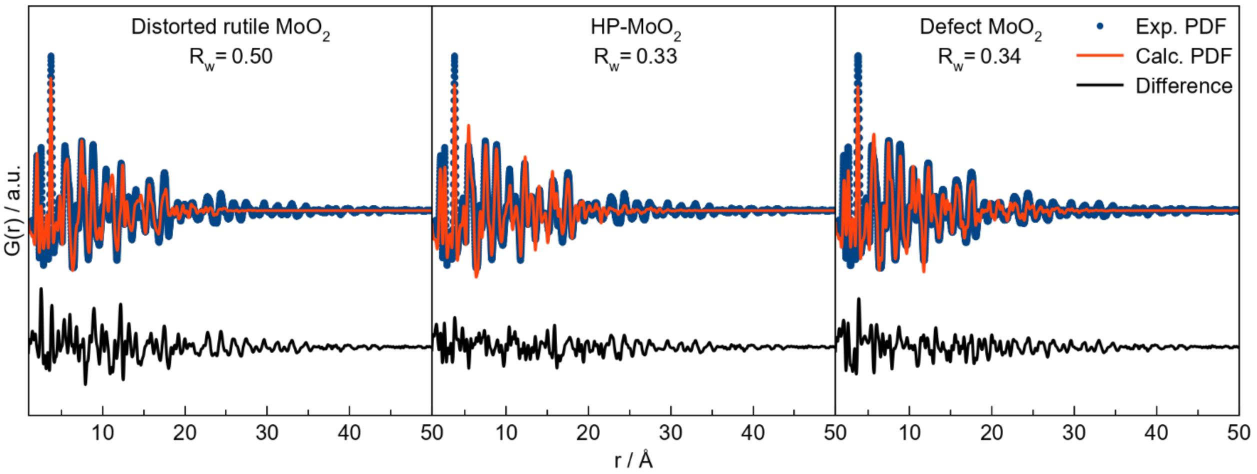

Figure S7. Real-space Rietveld refinement of molybdenum oxide formed *ex situ* in benzyl alcohol at 200 °C.

Table S12. Refinement parameters from real-space Rietveld refinement shown in Figure S7 left. Distorted rutile MoO_2 was used as the structural model, and the data was fitted in a range of 1 Å – 50 Å.

**Scale factor**	0.3635		
**Cell parameters [Å]**	a=5.7178	b=4.8693	c=5.5178
**Cell parameters [º]**		β=119.12	
**U_{iso} [Å^2]**	Mo=0.00599	O=0.00605	
**δ_2 [Å^2]**	3.6351		
**sp-diameter [Å]**	31.2561		
**R_w**	0.50		

Table S13. Refinement parameters from real-space Rietveld refinement shown in Figure S7 middle. HP-MoO_2 was used as the structural model, and the data was fitted in a range of 1 Å – 50 Å.

**Scale factor**	0.4513		
**Cell parameters [Å]**	a=9.8548	b=8.4492	c=4.7788
**U_{iso} [Å^2]**	Mo=0.00725	O=0.00711	
**δ_2 [Å^2]**	2.0		
**sp-diameter [Å]**	35.82		
**R_w**	0.33		

Table S14. Refinement parameters from real-space Rietveld refinement shown in Figure S7 right. Defect MoO_2 was used as the structural model, and the data was fitted in a range of 1 Å – 50 Å.

**Scale factor**	0.3541		
**Cell parameters [Å]**	a=5.6072	b=4.7622	c=5.7210
**Cell parameters [°]**		β=119.29	
**Defect Mo occupancy**	Mo=0.30		
**U_{iso} [$Å^2$]**	Mo=0.00619	O=0.0112	
**$δ_2$ [$Å^2$]**	3.6		
**sp-diameter [Å]**	38.912		
**R_w**	0.39		

Refinements of *ex situ* syntheses at 150 °C

Table S15. Fitted values obtained from Real-space Rietveld refinement of molybdenum oxide formed *ex situ* at 150 °C.

	Ethanol		Isopropanol		Benzyl alcohol	
	MoO_2	HP-MoO_2	MoO_2	HP-MoO_2	MoO_2	HP-MoO_2
R_w	0.50	0.48	0.49	0.43	0.48	0.40
Size (nm)	1.6	2.5	2.4	2.6	2.9	3.1

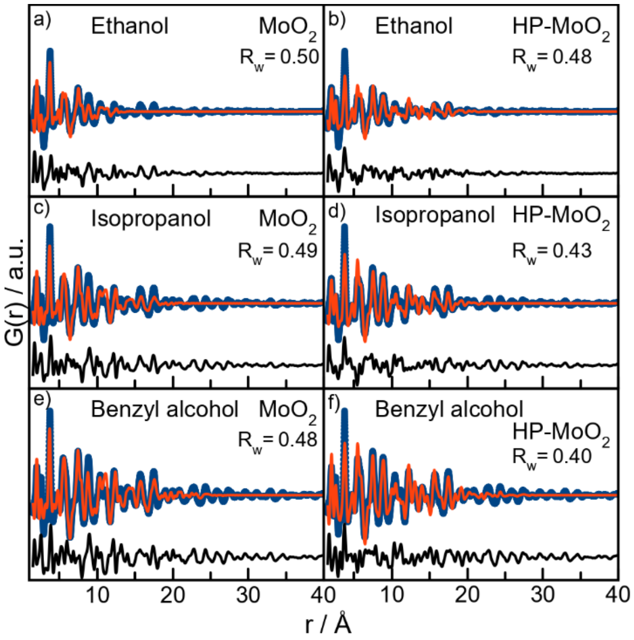

Figure S8. Real-space Rietveld refinement of molybdenum oxide formed *ex situ* in a,b) ethanol, c,d) isopropanol, and e,f) benzyl alcohol at 150 °C.

Table S16. Refinement parameters from real-space Rietveld refinement of molybdenum oxide formed *ex situ* in ethanol at 150 °C.shown in Figure S8a-b. Distorted rutile MoO_2 or HP-MoO_2 was used as the structural model, and the data was fitted in a range of 1 Å – 50 Å.

	**Distrorted rutile MoO_2**	**HP-MoO_2**
**Scale factor**	0.7575	0.6200
**Cell parameters [Å]**	a=5.7938 b=4.9188 c=5.4602	a=10.002 b=8.4006 c=4.7506
**Cell parameters [º]**	β=118.606	
**U_{iso} [Å^2]**	Mo=0.009 O=0.005	Mo= 0.009 O=0.005
**δ_2 [Å^2]**	2.0	3.18
**sp-diameter [Å]**	15.8	25.0
**R_w**	0.50	0.48

Table S17. Refinement parameters from real-space Rietveld refinement of molybdenum oxide formed *ex situ* in isopropanol at 150 °C.shown in Figure S8c-d. Distorted rutile MoO_2 or HP-MoO_2 was used as the structural model, and the data was fitted in a range of 1 Å – 50 Å.

	**Distrorted rutile MoO_2**	**HP-MoO_2**
**Scale factor**	0.7152	0.8938
**Cell parameters [Å]**	a=5.6620 b=4.8922 c=5.4726	a=9.8971 b=8.4499 c=4.7493
**Cell parameters [º]**	β=118.072	
**U_{iso} [Å^2]**	Mo=0.010 O=0.005	Mo=0.013 O=0.005
**δ_2 [Å^2]**	2.0	2.0
**sp-diameter [Å]**	24.02	25.98
**R_w**	0.49	0.43

Table S18. Refinement parameters from real-space Rietveld refinement of molybdenum oxide formed *ex situ* in benzyl alcohol at 150 °C.shown in Figure S8e-f. Distorted rutile MoO_2 or HP-MoO_2 was used as the structural model, and the data was fitted in a range of 1 Å – 50 Å.

	**Distrorted rutile MoO_2**			**HP-MoO_2**		
**Scale factor**	0.7529			0.9230		
**Cell parameters [Å]**	a=5.6599	b=4.8741	c=5.4913	a=9.8198	b=8.4810	c=4.7574
**Cell parameters [°]**		β=118.454		Mo=0.013	O=0.010	
**U_{iso} [Å^2]**	Mo=0.009	O=0.005		2.0		
**δ_2 [Å^2]**	2.0			30.99		
**sp-diameter [Å]**	29.12			0.40		
**R_w**	0.48			0.9230		

Examination of physical properties of the alcohols

Table S19. Physical properties and constants of methanol, ethanol, isopropanol, benzyl alcohol, and *tert*-butanol.[2-5] *Measured using Ag/Ag$^+$ as reference electrode.

	**Methanol**	**Ethanol**	**Isopropanol**	**Benzyl alcohol**	***tert*-butanol**
**M (g/mol)**	32.04	46.07	60.10	108.14	74.21
**T_m (°C)**	-97.65	-114.15	-89.55	-15.25	25.35
**T_b (°C)**	64.55	78.25	82.55	205.35	85.35
**T_c (°C)**	239.45	240.75	235.15	471.85	233.05
**P_c (bar)**	80.92	61.37	47.62	43.00	39.73
**E° ($V_{Fc/Fc+}$)**	2.73	2.61	2.50	>2.00*	2.60
**μ (D)**	1.70	1.69	1.58	1.71	1.64
**-$\chi_M \cdot 10^6$ (cm^3/mol)**	21.15	33.60	45.79	71.83	57.60
**p*K*a**	15.5	16.0	17.1	15.4	18.0
**ρ (g/mL)**	0.7914	0.7893	0.7855	1.0419	0.7887
**n (mol)**	0.240	0.171	0.131	0.096	0.106
**Pvap@25 °C(kPa)**	16.9	7.87	6.02	0.015	2.32
**E_S**	0	-0.07	-0.47	-0.38	-1.54

Calculated PDF

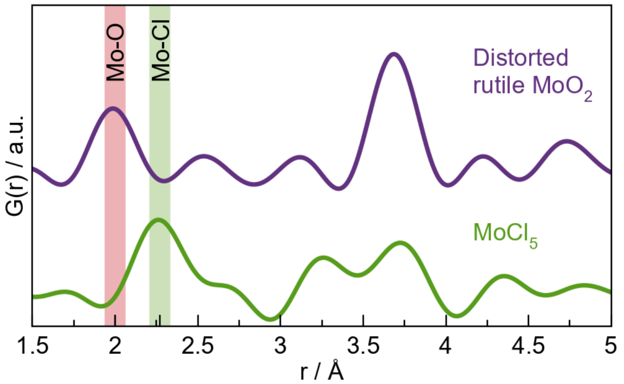

Figure S9. Calculated PDFs of distorted rutile MoO_2 (purple graph) and monoclinic $MoCl_5$ (green graph).[6] Mo-O distance at 2.0 Å is shown in red highlight, with Mo-Cl at 2.3 Å highlighted in green.

Investigation of the precursor solutions

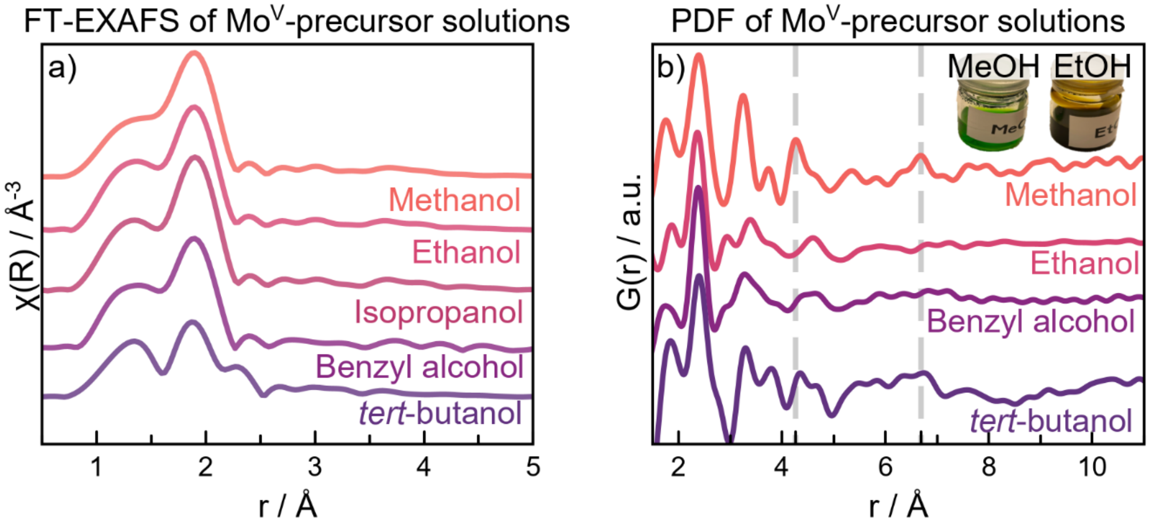

Figure S10. Investigation of the resulting precursor solutions using methanol, ethanol, isopropanol, benzyl alcohol, or *tert*-butanol as solvent mixed with $MoCl_5$. a) k^2 weighted EXAFS Fourier transform data. Phase shift uncorrected. b) PDF of precursor solutions using methanol, ethanol, benzyl alcohol, or *tert*-butanol as solvent. Insert of the resulting green solution obtained by mixing $MoCl_5$ and methanol for 1 minute, and brown solution by mixing $MoCl_5$ and ethanol for 1 minute.

Formation of distorted rutile MoO_2 in *tert*-butanol

Precursor fits of $MoCl_5$ in *tert*-butanol at RT

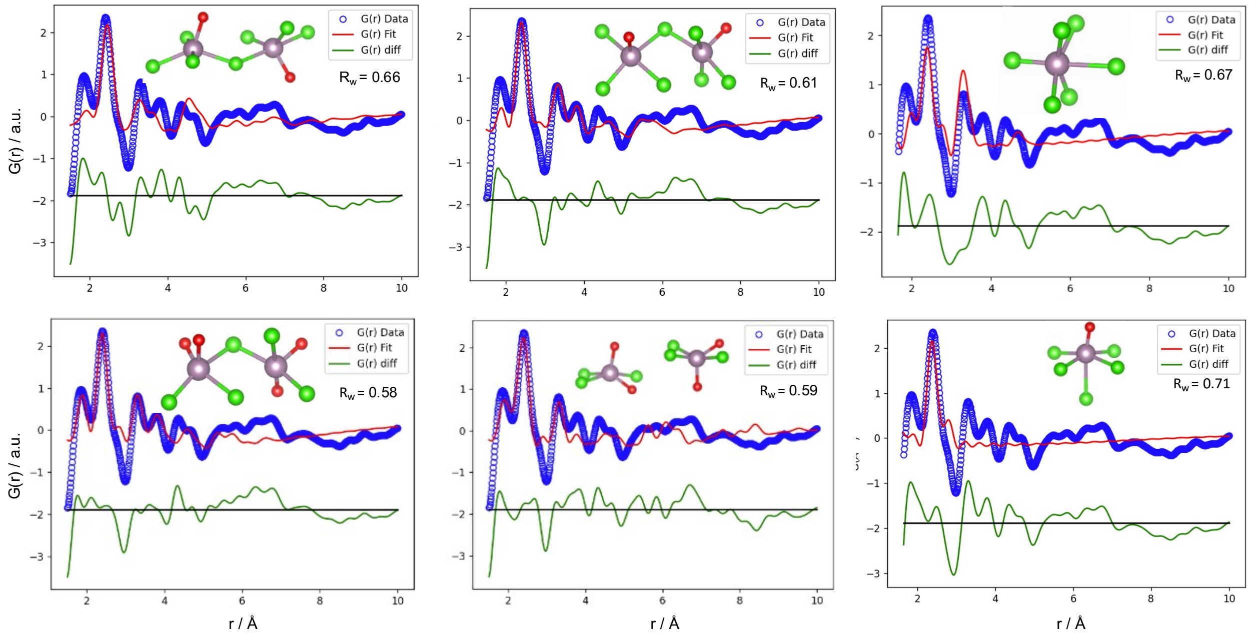

Figure S11. Real-space Rietveld refinements on selected structural motifs fitted to data obtained from $MoCl_5$ in *tert*-butanol at room temperature. Atoms in purple signify molybdenum, red is oxygen, and green is chloride. The best fits are obtained using a dimeric oxychloride species such as [$Mo_2Cl_5O_4$].

Cluster fits of intermediate species

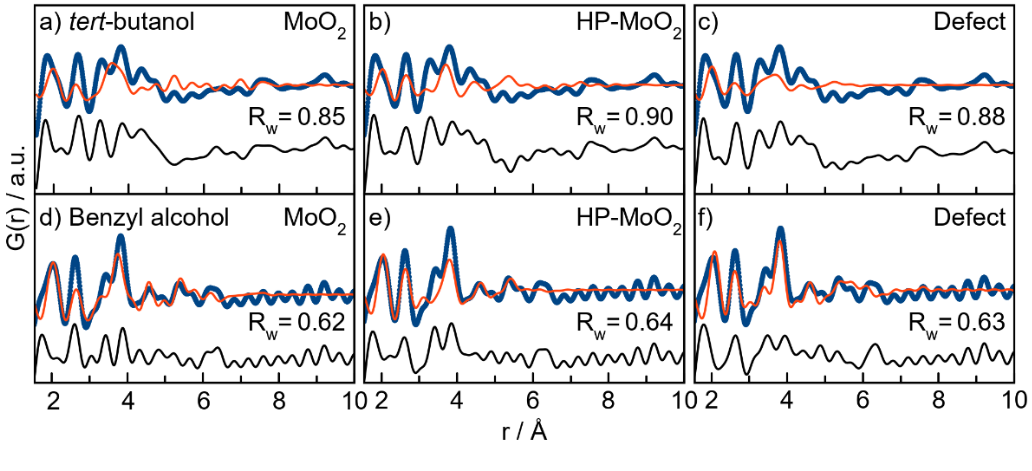

Figure S12. Real-space Rietveld refinements on the intermediate species formed after 1.5 min in a-c) *tert*-butanol and d-f) benzyl alcohol, at 200 °C. Fitted with a,d) distorted rutile MoO_2, b,e) HP-MoO_2, and c,f) defect structure.

Cluster fitting of the intermediate species formed *in situ* at 200 °C after 2 minutes of reaction time was carried out using clusters with different structural motifs cut out from the bulk distorted rutile MoO_2 structure. The used clusters are shown in Figure S13. The models were fitted to the PDFs in the range of 1.5 to 10 Å allowing refinement on the scale factor, lattice parameter, atomic position of Mo, and atomic displacement parameters (ADP) for both Mo and O atoms. The fit residual values, R_w, for each fitted intermediate species,

are summarized in Table S20. The lowest values of R_w obtained from the fitting of each solution are marked in bold.

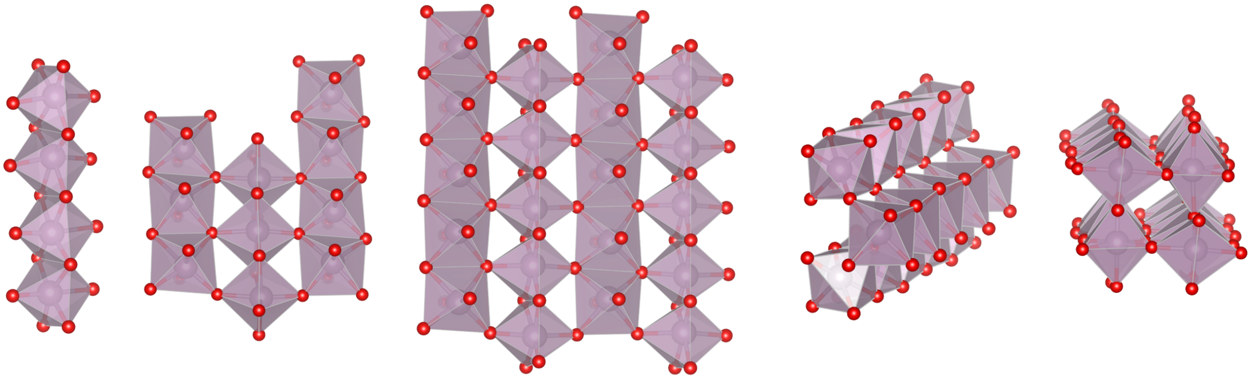

Figure S13. Clusters cut out from distorted rutile MoO_2 which are used as structural models for fitting intermediate clusters.

Table S20. R_w values for PDF intermediate cluster fit.

	4x1	4x3x1	4x5x1	5x2x1	5x2x2
tert-butanol 200 °C	0.90	0.82	**0.63**	0.87	0.89
Benzyl alcohol 200 °C	0.95	0.80	**0.49**	0.82	0.78

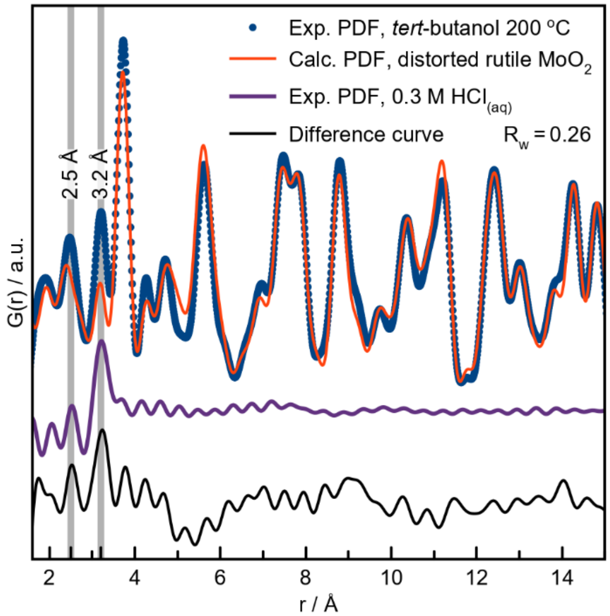

Figure S14. PDF of product obtained *in situ* using *tert*-butanol at 200 °C, overlayed an experimental PDF obtained from 0.3 M $HCl_{(aq)}$.

When examining the PDF fit of the product obtained *in situ* with *tert*-butanol heated to 200 °C using a distorted rutile MoO_2 model in Figure S14, we observe the peaks beyond 5 Å are well-described by the model. However, significant discrepancies are observed in the local range, as highlighted by the difference curve. This behavior contradicts the PDF measured of the dry sample prepared *ex situ* (Figure 3d). Interactions from ions in solution may contribute to the additional PDF signals that are not captured by the MoO_2 model. Literature suggests that the additional peak intensity at 3.2 Å could arise from a Cl-O hydration shell.[7, 8] As the chloride salt, $MoCl_5$, is used as the precursor, chloride interactions in the solvent are plausible. To test this hypothesis, we compare the difference curve with the experimental PDF of a 0.3 M HCl solution (Figure S14, purple line). The features of the HCl PDF closely match the experimental difference curve, as highlighted by the vertical grey lines. We attribute these characteristic distances to the presence of a chloride solvation shell in the solution.

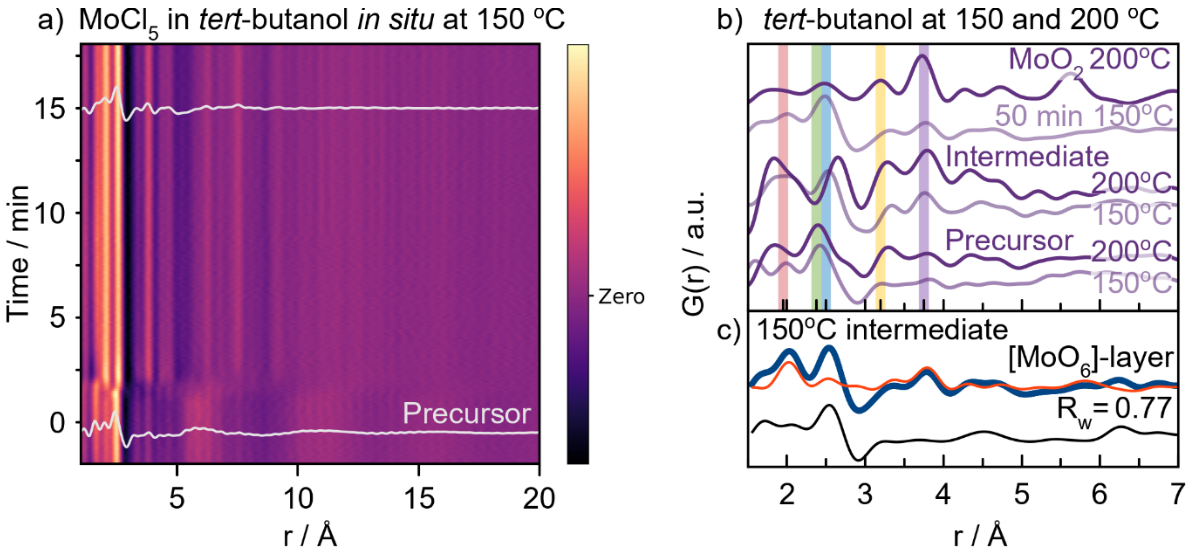

Figure S15. a) Time-resolved PDF from *in situ* synthesis using *tert*-butanol as solvent. Temperature is 150 °C. b) Comparison of selected PDFs measured *in situ* in *tert*-butanol at 150 and 200 °C. Colored highlights indicate significant distances: Mo-O (red), Mo-Cl (green), edge-sharing Mo-Mo (blue and yellow), and corner-sharing Mo-Mo (purple). c) Intermediate formed in *tert*-butanol at 150 °C fitted to the 4x5x1 cluster visualized in Figure 4f.

Formation of distorted rutile MoO_2 in benzyl alcohol

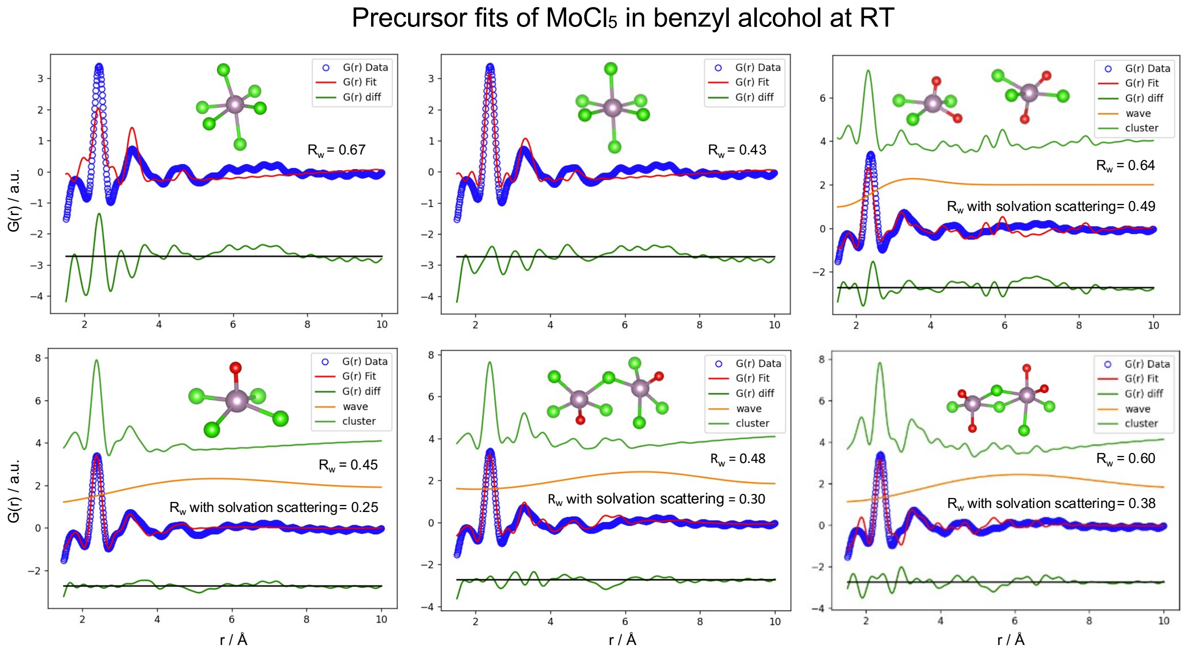

Figure S16. Real-space Rietveld refinements with selected structural motifs fitted to data obtained from $MoCl_5$ in benzyl alcohol at room temperature. Atoms in purple signify molybdenum, red is oxygen, and green is chloride. A broad wave-like feature is observed in this PDF, which is a result of a restructuring of the solvent around the cluster.[9] To account for scattering from solvent-shells a dampened sine-wave as described by Zobel et. al.[9] has been included in the fits. The best fits are obtained using a monomeric oxychloride species such as [$MoCl_4O$].

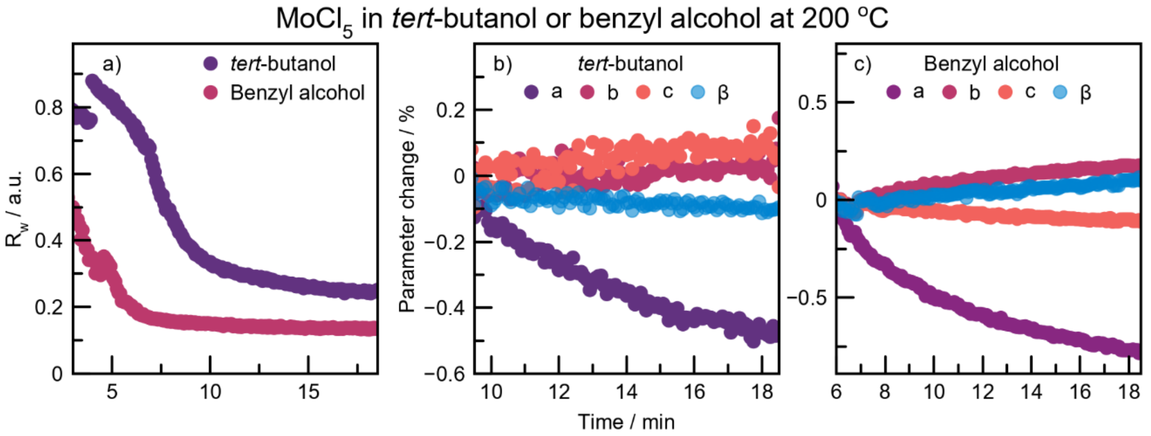

Figure S17. Parameters extracted from sequential PDF refinements from data obtained on $MoCl_5$ in *tert*-butanol (a-b) or benzyl alcohol (a,c) at 200 °C. Distorted rutile MoO_2 is used as the structural model. a) R_w, b-c) lattice parameters.

Formation of HP-MoO_2 in benzyl alcohol

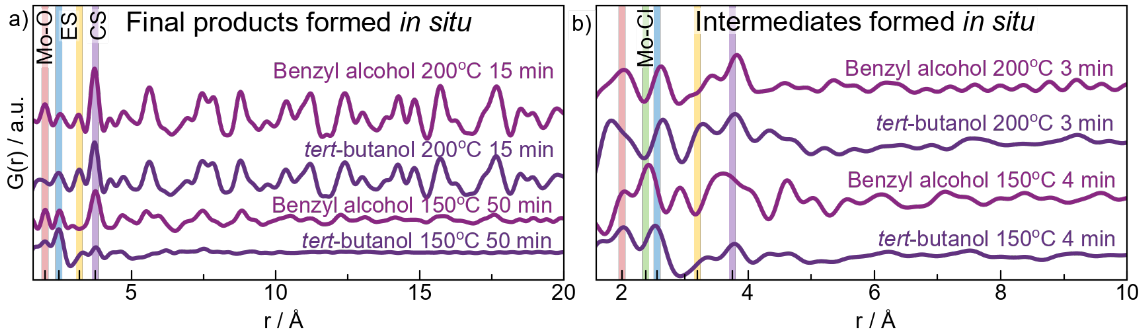

Figure S18. Comparison of PDFs measured *in situ* of a) final products and b) intermediate clusters, formed in *tert*-butanol and benzyl alcohol at 150 and 200 °C.

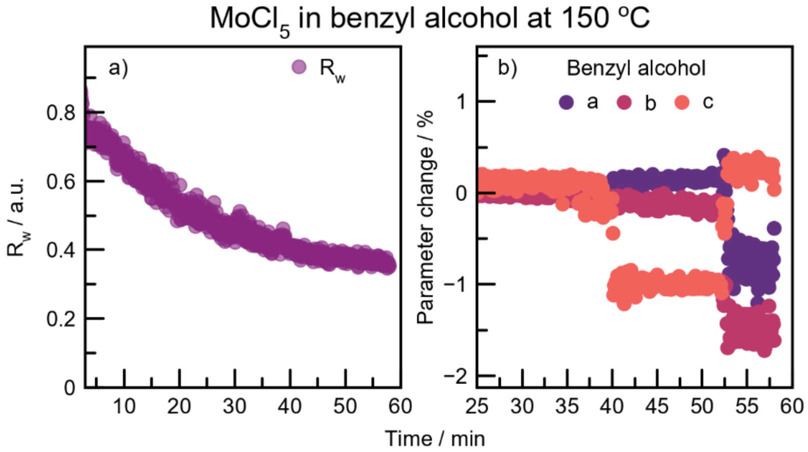

Figure S19. Parameters extracted from sequential PDF refinements from data obtained on $MoCl_5$ in benzyl alcohol at 150 °C. HP-MoO_2 is used as the structural model. a) $R_{w,}$ b) lattice parameters.

X-ray absorption spectroscopy

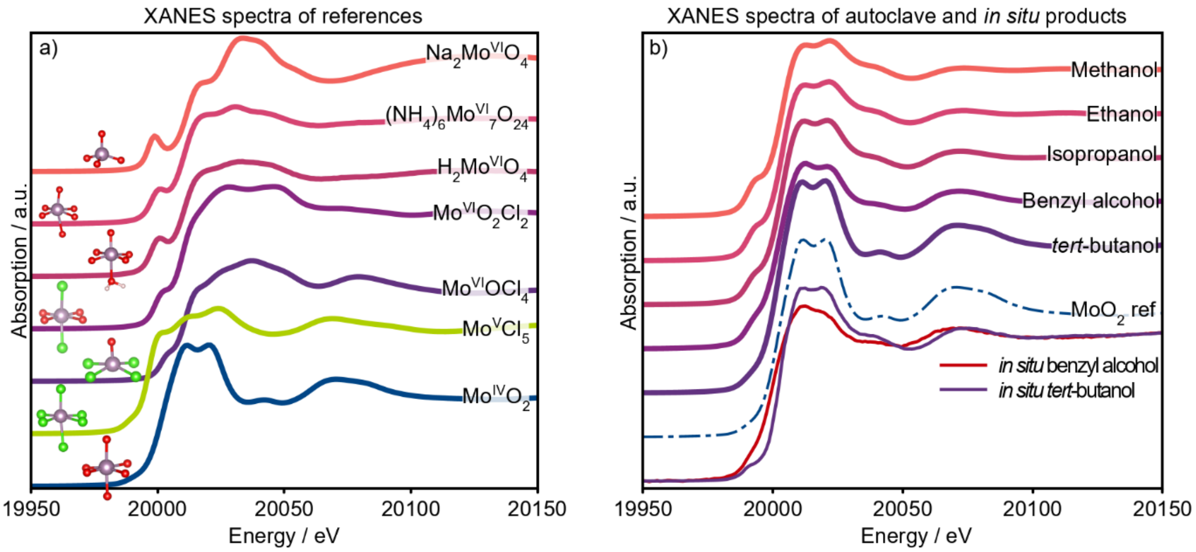

Figure S20. XANES spectra. a) Reference spectra collected on commercially obtained powders, with the local Mo coordination inserted. b) MoO_2 produced from the autoclave synthesis using five different solvents (fat lines). Blue dashed line shows the recorded XANES spectra of a commercial crystalline distorted rutile MoO_2. Thin graphs show recorded XANES spectra of *in situ* synthesized HP-MoO_2 using benzyl alcohol (red) or distorted rutile MoO_2 using *tert*-butanol (purple) as the solvent after heating to 150 °C.

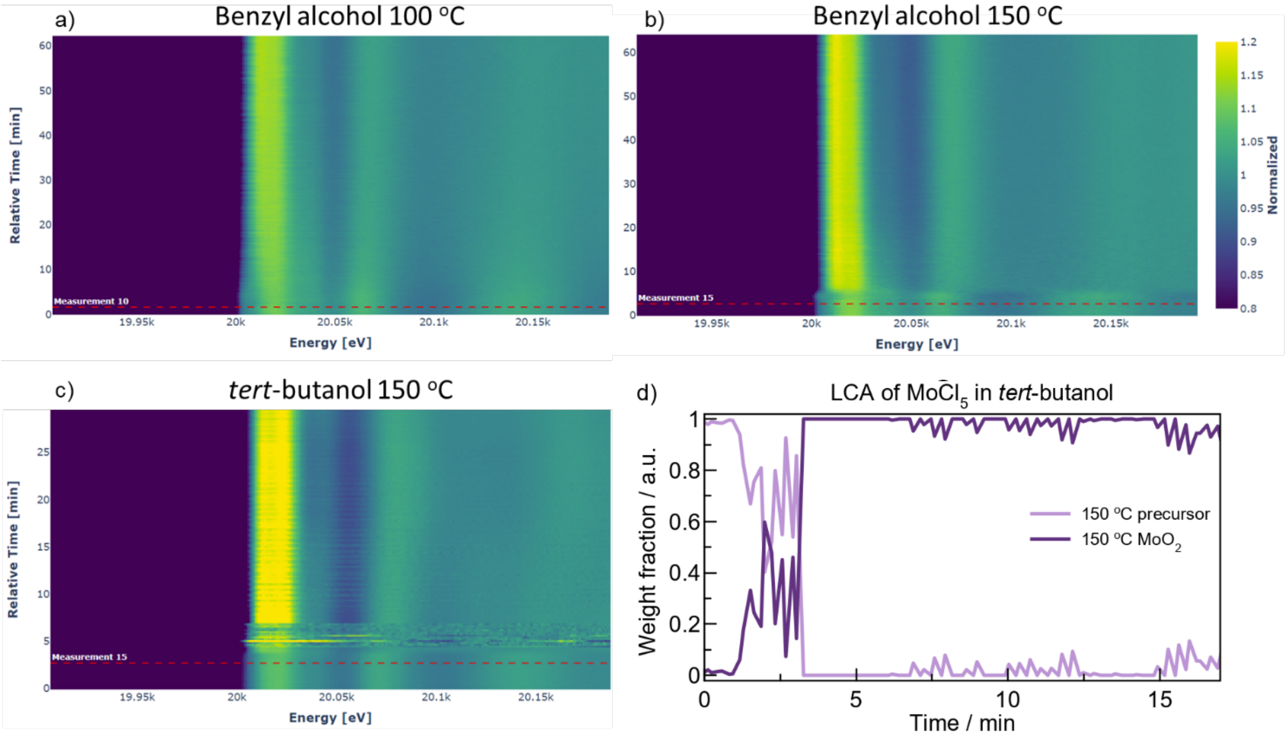

Figure S21. Time-resolved XANES spectra of $MoCl_5$ in a) benzyl alcohol at 100 °C, b) benzyl alcohol at 150 °C, and c) *tert*-butanol at 150 °C. To minimize beam damage, 7-second measurements alternated between three spots along the capillary. However, inconsistent heating caused by variations in the heating coils, as described in Figure S2, leads to irregular XANES patterns, which are particularly noticeable in c). d) Linear combination analysis (LCA) of the data shown in c). The measurement at t = 0 min was used as the precursor reference. Note that every third data point is excluded, due to significant differences in heating rates at one of the measurement spots compared to the other two.

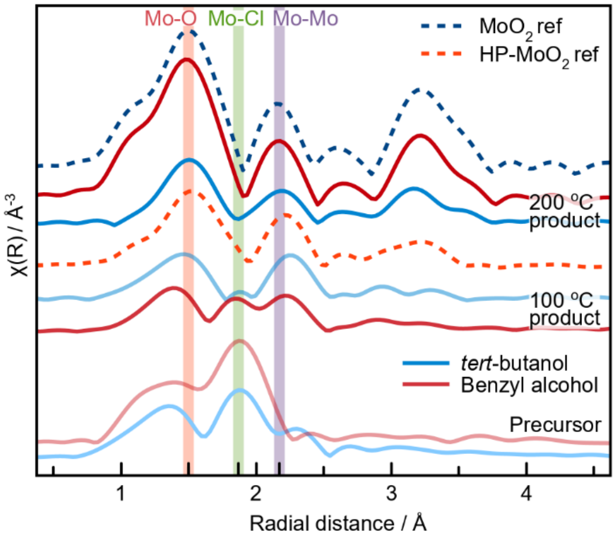

Figure S22. k^2 weighted EXAFS Fourier transform of precursor solutions and resulting products. Precursor solutions were measured at room temperature after mixing $MoCl_5$ in benzyl alcohol (pale red) or *tert*-butanol (pale blue). EXAFS Fourier transform of $MoCl_5$ in benzyl alcohol (light red) or *tert*-butanol (light blue) heated at 100 °C for 40 min. EXAFS Fourier transform of $MoCl_5$ in benzyl alcohol (red) or *tert*-

butanol (blue) heated at 200 °C for 20 min. Dashed lines represent the EXAFS Fourier transform of the *ex situ* autoclave synthesis at 200 °C in *tert*-butanol (dark blue) and benzyl alcohol (dark red). Phase shifts are uncorrected.

XAS study of the possible influence of trace water on Mo^{V} reduction

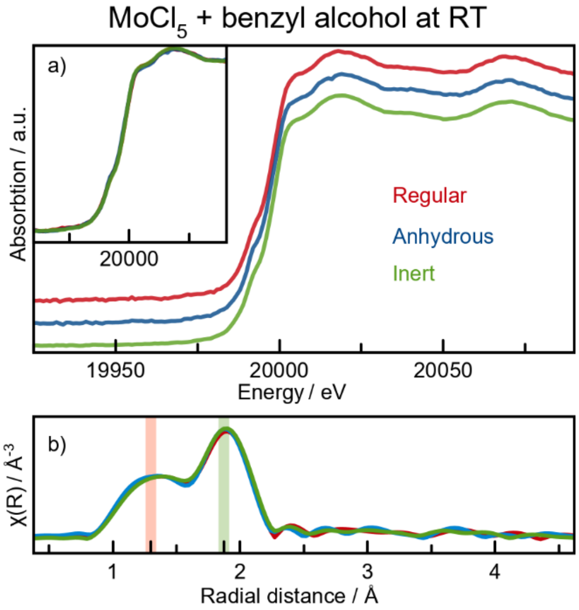

Figure S23. XAS measured of $MoCl_5$ in benzyl alcohol under various conditions at room temperature. Red graphs: atmospheric conditions (Sigma-Aldrich ≤ 0.10 % water). Blue graphs: anhydrous benzyl alcohol (Sigma-Aldrich, Anhydrous <0.005 % water) in atmospheric conditions. Green graphs: anhydrous benzyl alcohol in an inert atmosphere. a) XANES. Patterns are shifted 0.1 in y for clarity. The insert shows the three spectra plotted on top of each other. b) k^2 weighted EXAFS Fourier transform. Mo-O distance is shown in red highlight and Mo-Cl in green highlight, phase shift uncorrected.

PDF cluster fits of $MoCl_5$ in ethanol or methanol

Precursor fits of $MoCl_5$ in ethanol at RT

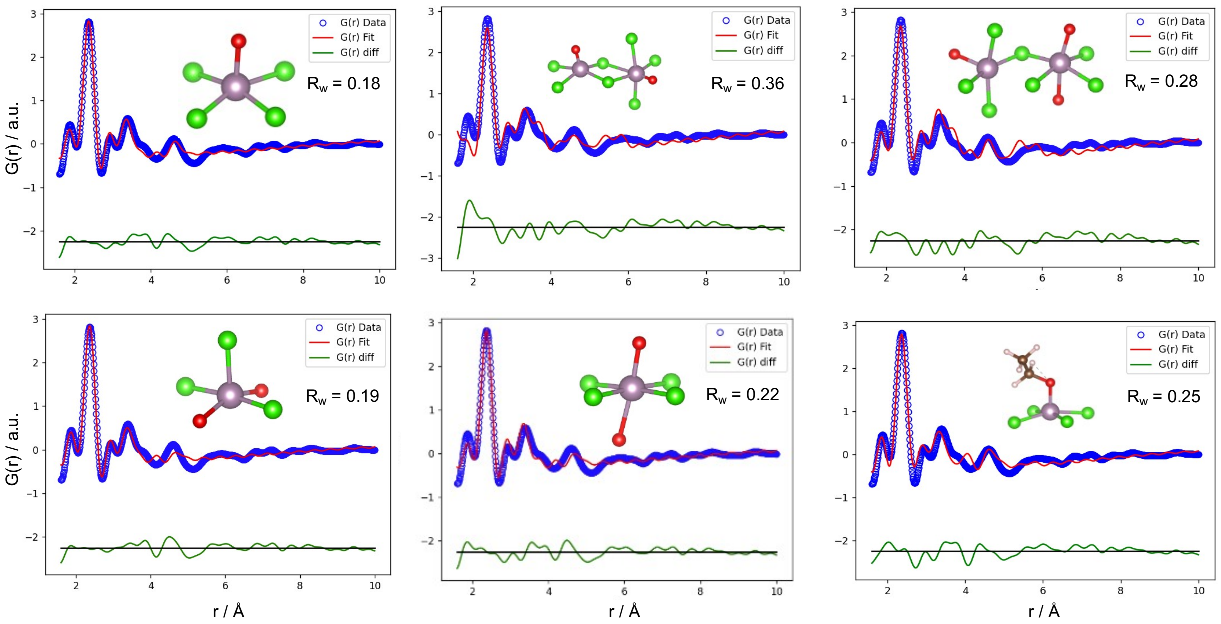

Figure S24. Real-space Rietveld refinements on selected structural motifs fitted to data obtained from $MoCl_5$ in ethanol at room temperature. Atoms in purple signify molybdenum, red is oxygen, green is chloride, brown is carbon, and hydrogen is white. The best fits are obtained using a monomer oxychloride species such as [$MoCl_4O$].

Precursor fits of $MoCl_5$ in methanol at RT

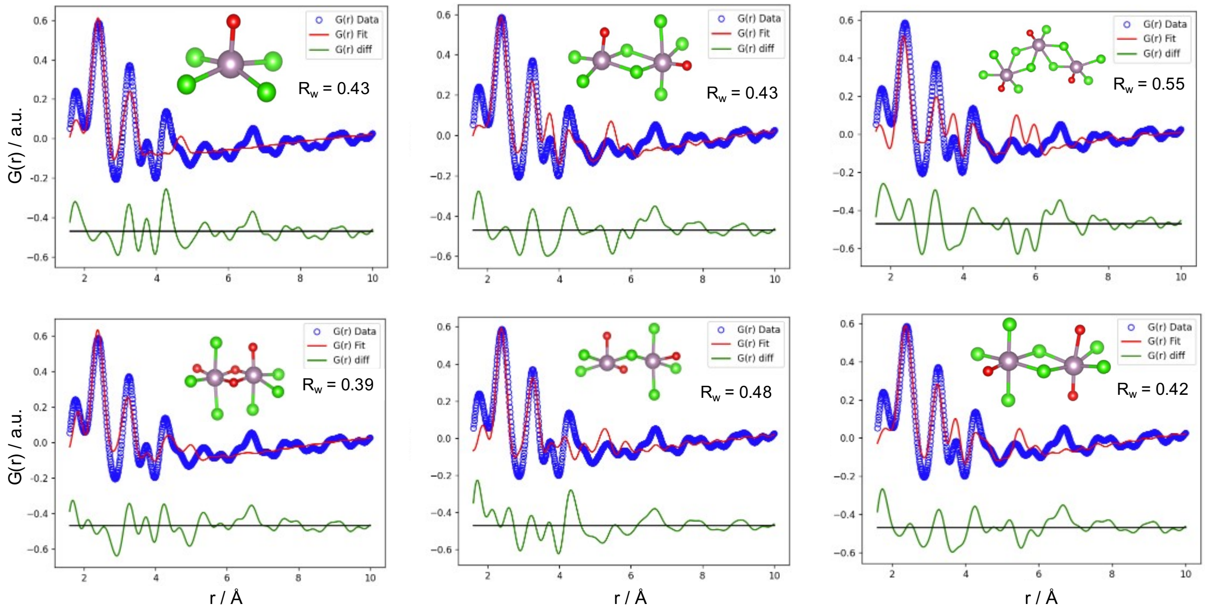

Figure S25. Real-space Rietveld refinements on selected structural motifs fitted to data obtained from $MoCl_5$ in methanol at room temperature. Atoms in purple signify molybdenum, red is oxygen, and is green chloride. The best fits are obtained using a dimeric oxychloride species such as [$Mo_2Cl_6O_4$].

Crystallographic Information Files reference codes

Table S21. Crystallographic Information Files (CIF) reference codes.

Structure	Ref. code	Reference
MoO_2	36263 (ICSD)	[10]
HP-MoO_2	243549 (ICSD)	[11]

References

1. T. L. Christiansen, E. D. Bøjesen, M. Juelsholt, J. Etheridge and K. M. Ø. Jensen, Size Induced Structural Changes in Molybdenum Oxide Nanoparticles, *ACS Nano*, 2019, **13**, 8725-8735.
2. D. R. Lide, *CRC Handbook of Chemistry and Physics*, CRC Press, 72 edn., 1991.
3. T. Fuchigami, S. Inagi and M. Atobe, *Appendix B: Tables of Physical Data*, John Wiley & Sons Ltd, 1 edn., 2015.
4. Phenols, Alcohols and Carboxylic Acids - pKa Values, https://www.engineeringtoolbox.com/paraffinic-benzoic-hydroxy-dioic-acids-structure-pka-carboxylic-dissociation-constant-alcohol-phenol-d_1948.html (accessed 24/03, 2025).
5. N. Trišović, N. Valentić and G. Uščumlić, Solvent effects on the structure-property relationship of anticonvulsant hydantoin derivatives: A solvatochromic analysis, *Chem. Cent. J.*, 2011, **5**, 62.
6. D. E. Sands and A. Zalkin, The crystal structure of $MoCl_5$, *Acta Crystallogr.*, 1959, **12**, 723-726.
7. H. S. Kim, J. Y. Koo and H. C. Choi, Mechanical Grinding Effect: Facile Sublimation and Its Application in Molecular Cocrystal Synthesis by Physical Vapor Transport, *Cryst. Growth Des.*, 2025, **25**, 171-175.
8. F. Bruni, S. Imberti, R. Mancinelli and M. A. Ricci, Aqueous solutions of divalent chlorides: Ions hydration shell and water structure, *J. Chem. Phys.*, 2012, **136**, 064520.
9. M. Zobel, R. B. Neder and S. A. J. Kimber, Universal solvent restructuring induced by colloidal nanoparticles, *Science*, 2015, **347**, 292-294.
10. A. Magnéli, The Crystal Structure of the Dioxides of Molybdenum and Tungsten, *Ark. Kemi. Mineral. Geol.*, 1947, **24**, 1-11.
11. T. Lüdtke, D. Wiedemann, I. Efthimiopoulos, N. Becker, S. Seidel, O. Janka, R. Pöttgen, R. Dronskowski, M. Koch-Müller and M. Lerch, HP-MoO_2: A High-Pressure Polymorph of Molybdenum Dioxide, *Inorg. Chem.*, 2017, **56**, 2321-2327.
